# Comprehensive genomic resources related to domestication and crop improvement traits in Lima bean

**DOI:** 10.1038/s41467-021-20921-1

**Published:** 2021-01-29

**Authors:** Tatiana Garcia, Jorge Duitama, Stephanie Smolenski Zullo, Juanita Gil, Andrea Ariani, Sarah Dohle, Antonia Palkovic, Paola Skeen, Clara Isabel Bermudez-Santana, Daniel G. Debouck, Jaime Martínez-Castillo, Paul Gepts, Maria Isabel Chacón-Sánchez

**Affiliations:** 1 Departamento de Agronomía, Facultad de Ciencias Agrarias, Universidad Nacional de Colombia, Bogotá, Colombia; 2 Systems and Computing Engineering Department, Universidad de los Andes, Bogotá, Colombia; 3 Department of Plant Sciences/MS1, University of California, Davis, CA USA; 4 Departamento de Biología, Facultad de Ciencias, Universidad Nacional de Colombia, Bogotá, Colombia; 5 Centro Internacional de Agricultura Tropical, Cali, Colombia; 6 Centro de Investigación Científica de Yucatán, Yucatán, Mexico; 7 Present Address: Biochemistry and Molecular Biology, Michigan State University, East Lansing, MI USA; 8 Present Address: Department of Entomology and Plant Pathology, University of Arkansas, Fayetteville, AR USA; 9 Present Address: BASF BBCC - Innovation Center, Gent, Belgium; 10 Present Address: Nunhems USA, Vegetable Seeds BASF, Acampo, CA USA

**Keywords:** Genetic variation, Agricultural genetics, Genome evolution, Plant domestication

## Abstract

Lima bean (*Phaseolus lunatus L*.), one of the five domesticated *Phaseolus* bean crops, shows a wide range of ecological adaptations along its distribution range from Mexico to Argentina. These adaptations make it a promising crop for improving food security under predicted scenarios of climate change in Latin America and elsewhere. In this work, we combine long and short read sequencing technologies with a dense genetic map from a biparental population to obtain the chromosome-level genome assembly for Lima bean. Annotation of 28,326 gene models show high diversity among 1917 genes with conserved domains related to disease resistance. Structural comparison across 22,180 orthologs with common bean reveals high genome synteny and five large intrachromosomal rearrangements. Population genomic analyses show that wild Lima bean is organized into six clusters with mostly non-overlapping distributions and that Mesomerican landraces can be further subdivided into three subclusters. RNA-seq data reveal 4275 differentially expressed genes, which can be related to pod dehiscence and seed development. We expect the resources presented here to serve as a solid basis to achieve a comprehensive view of the degree of convergent evolution of *Phaseolus* species under domestication and provide tools and information for breeding for climate change resiliency.

## Abstract (web summary)

Lima bean is an important crop for improving food security in Latin America and elsewhere. Here, the authors assemble its genome, conduct population genomics analysis using genotyping-by-sequencing data, and identify differentially expressed genes between two pod developmental stages.

## Introduction

The *Phaseolus* genus comprises more than 70 species, of which five have been domesticated, namely *P. acutifolius* A. Gray (tepary bean), *P. coccineus* L. (ayocote or runner bean), *P. dumosus* Macfady (num, piloy, or year bean), *P. lunatus* L. (Lima bean), and *P. vulgaris* L. (common bean)^1^. Lima bean and common bean are the two agronomically and economically most significant species within the *Phaseolus* genus^2–4^. Lima bean provides a vital source of nutrients globally; its seeds contain at least 20% protein, more than 50% carbohydrates; it is a rich source of amino acids such as tryptophan, lysine, methionine, phenylalanine, threonine, valine, isoleucine, and leucine^5,6^.

Wild and domesticated Lima beans are found in a wide variety of climatic conditions along with their range of distribution from northern Mexico to northern Argentina^7–9^. Wild Lima beans are structured into three major gene pools according to genomic data^10^: two Mesoamerican (MI and MII) and one Andean (AI) gene pool. While MI occurs mainly in central-western Mexico, MII is more widely distributed from southern Mexico and Central America to tropical South America. The Andean gene pool AI is restricted to southern Ecuador and northern Peru, where this species apparently originated^11^. The possible existence of another Andean gene pool, the AII gene pool restricted to the Andes in central Colombia has been proposed, although this requires further confirmation. At least two domestication processes took place in Lima bean, one in Mesoamerica and one in the Andes^10,12–17^. The Andean domestication occurred from gene pool AI and gave rise to the Andean varieties characterized by large and flat seeds (Big Lima cultivars). The second event occurred in central-western Mexico from gene pool MI and gave rise to the Mesoamerican varieties characterized by having small rounded or oval-shaped seeds (Potato and Sieva cultivars, respectively)^18,19^. Lima bean is a good example of convergent evolution since both Mesoamerican and Andean landraces evolved similar traits under domestication, mainly larger pods and seeds, reduction or loss of pod dehiscence, loss of seed dormancy, determinate growth habit and reduced content of antinutritional seed compounds, among others.

Lima bean is a species of great interest for evolutionary research not only because it provides an opportunity to study the molecular basis of convergent phenotypic adaptation, but also because it shows adaptation to a wider range of ecological conditions compared to common bean^9^, especially to heat and drought stresses, traits that are key in scenarios of adaptation to climate change^20^. For these reasons, it is important to document the domestication process in Lima bean, since a good understanding of the phenotypic adaptations involved and their genetic control may lead to the identification of the related genes and alleles and efficient use of these alleles for future breeding activities. Lima bean genetic research has previously relied on the common bean reference genomes^21–24^. Lima and common bean are both diploid species (2n = 2x = 22 chromosomes; DNA: ~622 Mbp/1 C) with high levels of homozygosity throughout their genomes because of predominant autogamy^25^. Previous cytogenetic research confirms a high degree of synteny between the two species^26^. However, relying on a *P. vulgaris* genome alone may have consequences for downstream diversity analyses due to reference bias, produces loss of information and could even be misleading for predictions of genomic loci related to traits. The development of a whole-genome reference sequence for *P. lunatus* would provide a groundbreaking genetic resource for Lima bean research, while highlighting genomic differences among its domesticated relatives^25–29^.

In this research, we describe a large collaborative effort to provide high-quality genomic resources for Lima bean genetics and breeding. These include a high-quality reference genome, gene expression information in different tissues, accessions and developmental stages, and the most comprehensive assessment of genomic variability in a sample of close to 500 wild and domesticated accessions. By combining approaches based on comparative genomics and population genetics, we reveal a complete view of the distribution of genomic variability across the species and its relationship with the common bean. Moreover, we provide gene functional annotations and genetic loci associations for different traits relevant to domestication processes and breeding in Lima beans.

## Results

### A chromosome-level high-quality assembly for Lima bean

**Fig. 1:**
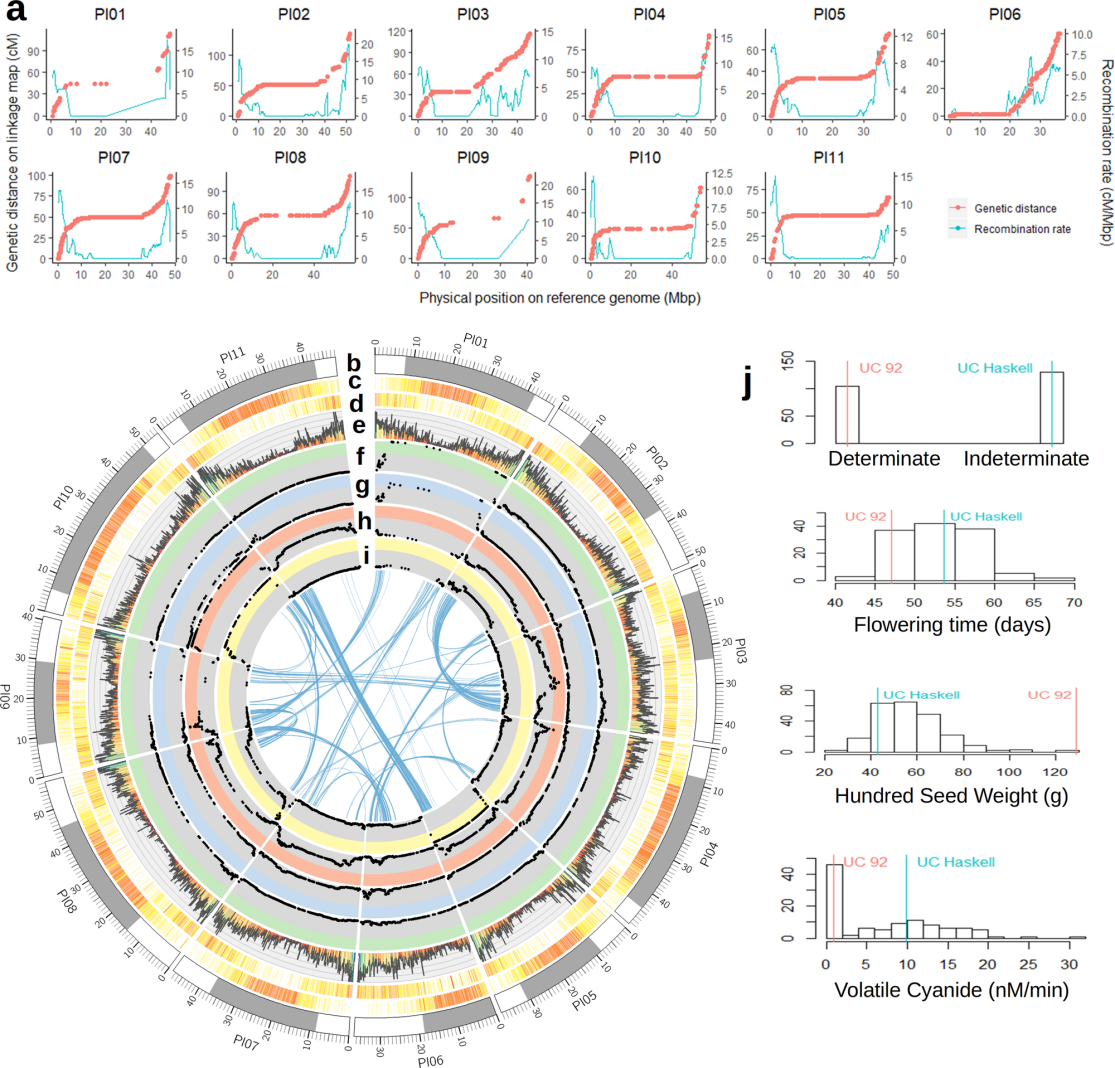
Chromosome-level genome assembly for Lima bean. **a** Genetic distance (cM) and recombination rate (cM/Mbp) by physical position (Mbp) on the Lima bean reference genome for the UC 92–UC Haskell RIL population. Chromosomes are labeled as Pl01-Pl11. **b** Chromosome lengths and pericentromeric regions. **c** Density of repetitive elements. **d** Density of gene models. **e** Density of SNPs. **f**–**i**. LOD scores of QTL for four different traits screened in the RIL population. Colored zones represent LOD scores greater than 3 for determinacy (green), flowering time (blue), hundred seed weight (red), and volatile cyanide (yellow). **j** Phenotypic distribution of traits in the RIL population with parental phenotypes represented by vertical lines. Source data are provided as a Source Data file.

We generated a chromosome-level assembly of the Lima bean genome from G27455, a domesticated accession from the Mesoamerican gene pool MI collected in northern Colombia. Data from the use of Pacific Biosciences and Illumina sequencing technologies and four experimental protocols, namely paired-end whole genome sequencing (WGS), 10x, genotyping-by-sequencing (GBS), and RNA sequencing, were combined to achieve the contiguity and base quality of this assembly. An initial backbone assembly of PacBio WGS reads polished using paired-end Illumina reads produced a draft assembly with 512 contigs adding up to 542 Mbp. A total of 206 of these contigs—totaling 512 Mbp—were further assembled in scaffolds, sorted, and oriented in 11 pseudomolecules based on an analysis of linked reads obtained from the G27455 accession following the 10x protocol and analysis of GBS data from the F_8_ generation of UC 92–UC Haskell recombinant inbred line population. A linkage map was developed for this population with 10,497 SNPs across 522 unique loci with an estimated genetic length of 1064 cM (Supplementary Fig. 1). Linkage groups were established for each of the 11 chromosomes (Supplementary Fig. 2 and Supplementary Table 1). This linkage map had an average genetic and physical spacing between loci of 2.18 cM and 1.10 Mbp, respectively. Genetic gaps larger than 20 cM were observed on three linkage groups: Pl01, Pl02, and Pl09, with 20.5, 24.4, and 32.8 cM gaps, respectively. Marker coverage varied across and within linkage groups with the densest marker coverage observed in the pericentromeric regions of Pl02, Pl05, Pl07, and Pl11, and the sparsest marker coverage observed in the pericentromeric regions of Pl01 and Pl09. Recombination rates varied within linkage groups, with the lowest rates of recombination in the centromeric and pericentromeric regions and the highest rates towards the telomeric ends (Fig. 1a and Supplementary Table 2). The Pl03 linkage group had the highest average recombination rate, with recombination events occurring every 662 kbp. The Pl10 linkage group had the lowest average recombination rate, with recombination events occurring on average every 2074 kbp, which may be influenced by the high degree of segregation distortion observed in the pericentromeric region towards the UC Haskell haplotype. The largest spans of the pericentromeric regions were on the Pl04, Pl10, and Pl11 linkage groups, and the shortest spans were on Pl03 and Pl06 (Fig. 1b and Supplementary Table 2). The Pl01 and Pl09 linkage groups had particularly sparse marker coverage across the pericentromeric regions, which likely reduced the accuracy of the recombination rates in these regions and the definition of the pericentromeric regions for these linkage groups.

Base-pair-level quality was assessed by the mapping of Illumina reads to the assembly, which reached 99% of the raw reads after polishing. In addition, the identification of orthologs reached 98.8% of 1614 genes known to be conserved in a single copy across plant species (Supplementary Fig. 3). Annotation of repetitive elements was performed using repeat masker^30^ and based on a common bean library of 796 transposable elements^31^. A total of 656,928 events were identified covering 225 Mbp (41% of the assembly). More than half of these regions (174 Mbp) were covered by long terminal repeats (LTRs). Additional 8 Mbp were covered by other class I retrotransposons, namely LINE and SINE elements. DNA (Class II) transposons covered 25 Mbp of the assembly and other 6 Mbp was covered by other unclassified transposable elements (Supplementary Table 3). Repetitive elements are more abundant within pericentromeric regions of the genome (Fig. 1c).

To perform automated structural and functional annotation of gene models for the Lima bean genome, repeat elements were masked and Illumina RNA-seq data from three tissues (leaf, flower, and pod), two developmental stages (initiation of pod elongation and before seed filling), and two accessions (G25230: wild from Manzanillo, Colima, Mexico; and G27455) were analyzed. In contrast to repetitive elements, gene density and observed number of SNPs is higher outside pericentromeric regions (Fig. 1d, e). By integrating publicly available RNA-seq data and gene models from the common bean genome, a total of 28,326 gene models and 35,881 transcripts were predicted with an average total length of 3.7 kbp, and an average protein length of 413 amino acids. Distributions of gene and protein length are consistent with the current gene annotation for *P. vulgaris* (Supplementary Fig. 4). Gene ontology functional annotations could be recognized for 21,642 (76%) of the gene models by ortholog identification from other plant species. Common ontology terms included response to stress, different metabolic processes, transport, anatomical structure development, signal transduction, cellular component assembly, and homeostasis (Supplementary Fig. 5). A total of 19,554 gene models were annotated with pathways from the Kyoto Encyclopedia of Genes and Genomes (KEGG). In total, functional annotations were assigned to 22,634 (80%) gene models. Gene expression was evidenced in at least one RNA-seq dataset for 26,295 (93%) gene models. Moreover, orthologs with *P. vulgaris* within synteny blocks were identified for 22,180 (78%) gene models (details in the next section). Considering only gene expression and orthology with *P. vulgaris* in synteny blocks, direct evidence could be identified for 27,029 (95%) of the annotated gene models. From the remaining gene models, 416 show at least 50% protein sequence similarity with *P. vulgaris* genes outside synteny blocks. The remaining 3% are either paralogs of genes with direct evidence or have functional annotations from other plant species.

### QTL mapping of agronomic-related traits in Lima bean

Quantitative trait loci (QTL) were mapped in the biparental population UC Haskell–UC 92 used to build the genetic map for genome assembly (Fig. 1f–i and Supplementary Table 4). Determinacy and three quantitative traits (flowering time, FT; hundred-seed weight, HSW; and cyanide content) were screened in this population (Fig. 1j). Nine quantitative trait loci (QTL) were identified in the biparental population. One major QTL for determinacy was identified on the long arm of chromosome Pl01, explaining 78% of the phenotypic variation. The peak LOD score for determinacy was located at the first marker on the long arm, after a nearly 20 cM and 20 Mbp gap in the pericentromeric region of chromosome Pl01. A likely causative gene for this locus is an ortholog of the *Arabidopsis* gene *TFL1*. The common bean ortholog *PvTFL1y* was previously mapped at 45Mbp of chromosome Pv01^32,33^. We identified the ortholog *PlTFL1* in Lima bean at 41Mbp of Pl01 (Supplementary Data 1). For flowering time, transgressive segregation was observed for both earlier and later progenies than the UC 92 and UC Haskell parents, respectively (Fig. 1j). A major QTL was also found on chromosome Pl01 and it is likely that the causative gene for this QTL is also *PlTFL1*. However, this QTL explained only 30% of the phenotypic variation, which suggests that other genes influence flowering time in this population.

Regarding seed weight, transgressive segregation was observed for seed weights below the small-seeded parent UC Haskell, but not for larger seed weights. This observation is consistent with prior results showing a shift towards smaller-seeded segregants, observed in common bean^34,35^. Four additive minor QTL were identified collectively explaining 28% of the phenotypic variation, including one on the long arm of chromosome Pl10 that explained 11% of the phenotypic variation. Finally, a major QTL for cyanogenesis in floral bud tissue was found on the long arm of chromosome Pl05 and explained 93% of the phenotypic variation, and collectively with two other minor QTL explained 97.5% of the total phenotypic variation for cyanogenesis. The UC 92 variety did not show measurable cyanide content in contrast with UC Haskell. There was transgressive segregation for cyanide content above the levels observed in UC Haskell. The three QTL showed epistatic interactions: the UC 92 allele of the larger QTL on Pl05, causal of the absence of cyanogenic glucosidase, prevented the expression of the two QTLs on Pl08 and Pl10 (Supplementary Fig. 6). The significance interval of the QTL for cyanide content on Pl05 includes a sequence for a glucosidase with homology to a cyanogenic glucosidase in white clover^36,37^.

### Evolution of paralogs and orthologs and speciation events

**Fig. 2:**
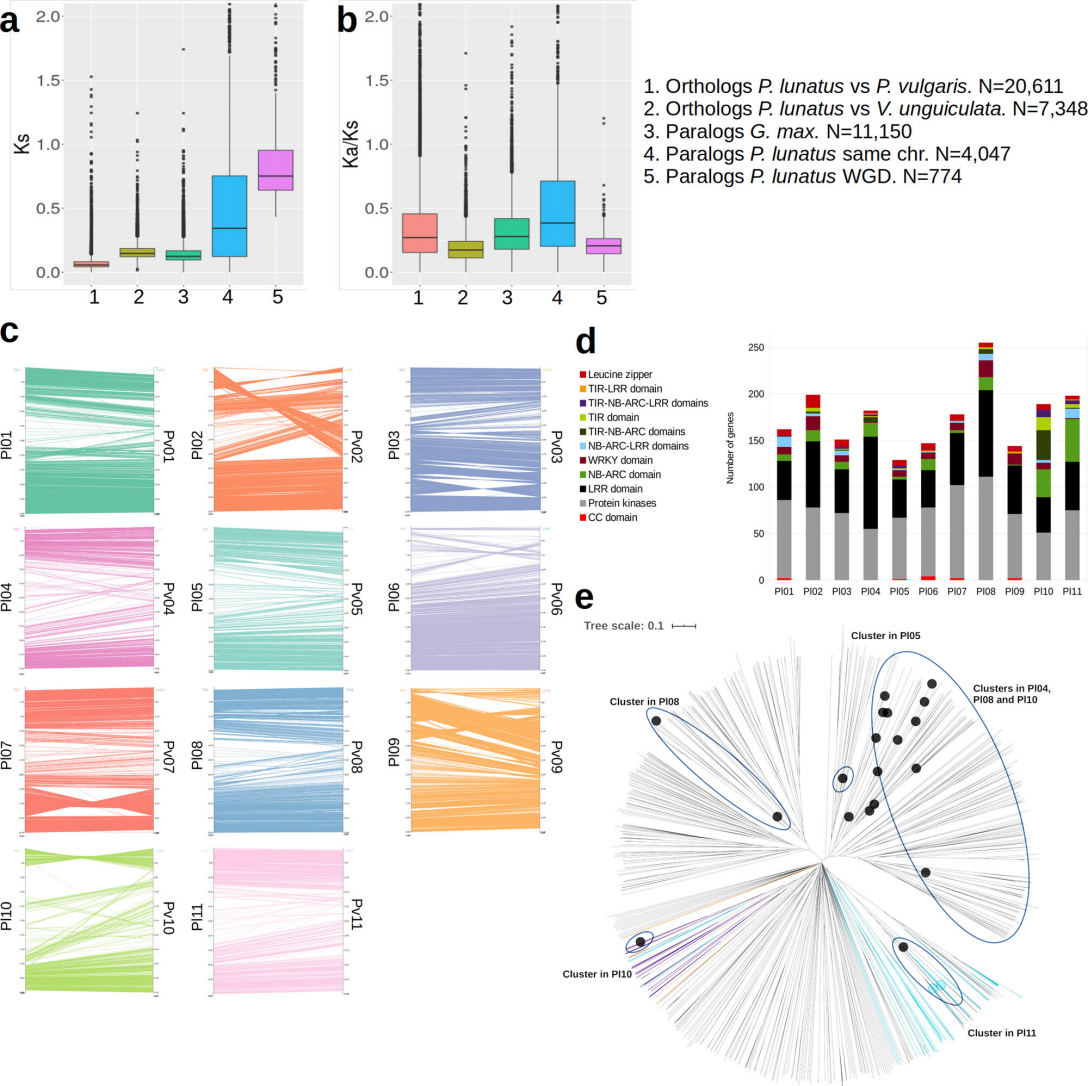
Comparative genomics between *P. lunatus* and *P. vulgaris*. **a** Ks and **b** Ka/Ks statistics for *P. lunatus* and *G. max* paralogs, as well as orthologs between *P. lunatus* and *P. vulgaris* and orthologs between *P. lunatus* and *V. unguiculata*. WGD: Whole Genome Duplication. Sample sizes (N) correspond to gene pairs. Middle lines are medians and box limits represent first (Q1) and third (Q3) quartiles. Lines are drawn from Q1 minus 1.5 of the interquartile range (IQR) to Q3 + 1.5*IQR. **c** Chromosome by chromosome synteny between *P. lunatus* and *P. vulgaris* for detailed visualization of structural rearrangements. *P. lunatus* chromosomes are labeled as Pl01-Pl11 and *P. vulgaris* chromosomes are labeled as Pv01-Pv11. **d** Number of homologs of resistance genes by chromosome. **e** NJ Radial tree diagram showing genetic variability among LRR type resistance genes. Light blue is proteins with domains NB-ARC and LRR, purple is proteins with domains TIR, NB-ARC, and LRR, orange is proteins with the TIR and LRR domains. Source data are provided as a Source Data file.

Predicted proteins for representative transcripts of all annotated genes were aligned to each other to build 3499 paralog clusters representing the gene families generated through different genome evolution processes. Classification of paralog relationships and interchromosomal synteny analysis revealed 1647 genes with paralogs generated by the ancient whole-genome duplication events documented in the history of Fabaceae^38^. Chromosome pairing inferred from these paralogs is consistent with that reported in the genome of *P. vulgaris*^23^ (Internal links in Fig. 1). Intrachromosomal duplication events were identified for a total of 7285 genes. Even after removing highly repeated genes (with more than ten paralogs), 5849 genes were involved in intrachromosomal duplication events. Figure 2a shows that the Ks values for these cases are significantly smaller than those of whole-genome duplication (WGD) paralogs (*p*-value < 10^−15^ for a Wilcoxon rank test), meaning that intrachromosomal duplications are more recent than WGD paralogs. Protein evolution among the two types of paralogs was assessed by calculating the Ka/Ks ratio between pairs of paralogs to identify patterns of selection. In contrast to Ks values, Ka/Ks values of intrachromosomal duplications are significantly larger than those of WGD paralogs (*p*-value < 10^−15^ for a Wilcoxon rank test), which means that these duplications are diverging faster than WGD paralogs (Fig. 2b). Moreover, 12% of the local duplications seem to experience a rapid sequence divergence showing Ka/Ks ratios above 1. Functional enrichment of 368 genes involved in recent duplications with high Ka/Ks ratios revealed a relatively large set of interconnected biological processes mostly related to immune response, including cell death, cell communication, and signaling (Supplementary Fig. 7). These findings are consistent with those reported by Qiao et al.^38^ for other plant species. Other processes enriched in evolving intrachromosomal duplications include lipid transport and metabolism of lignan.

We compared the genome of Lima bean assembled in this study with that of common bean v1.0^23^ based on the identification of orthologs between the two species and synteny blocks. Orthologs could be identified for 25,564 (94%) of the *P. vulgaris* genes and 26,009 (92%) of the *P. lunatus* genes. As reported in previous studies^26^, a high collinearity was observed between the *P. lunatus* and *P. vulgaris* genomes (Fig. 2c). The most important structural events identified in this study are an inversion of the short arm of chromosome Pl10 and a large translocation of the pericentromeric region of Pv02 within the short arm of chromosome Pl02. Other large events include a 5Mbp inversion within the long arm of chromosome Pl03, a 10Mbp inversion within the long arm of chromosome Pl07, and a complex translocation within the short arm of chromosome Pl09. The rearrangements in Pl02 and Pl10 confirm previous works suggesting pericentromeric inversions in these chromosomes based on Fluorescence in situ hybridization (FISH) assays^28^. As seen in other species, some of these rearrangements could be related to the previously observed reproductive isolation between Lima bean and common bean^39,40^.

Figure 2a shows that the Ks distribution of 22,180 orthologs between *P. lunatus* and *P. vulgaris* identified in synteny blocks is centered around 0.05. This is about half of the average obtained for paralogs generated by the recent (about 13 MYA) WGD event within the *G. max* genome^41^, suggesting that the speciation between *P. vulgaris* and *P. lunatus* occurred around 6 MYA. This date is close to the age of the *Phaseolus* crown clade B (that contains *P. vulgaris* and *P. lunatus)* estimated on the basis of evolutionary rates of the chloroplast *trnK* locus (5.0 ± 0.7 MYA) and older than the date estimated from ITS/5.8 S sequences (3.4 ± 0.4 MYA)^2^. Comparing the genomes of Lima bean and *Vigna unguiculata*^42^, the separation of *Phaseolus* from *Vigna* could be dated right before the WGD of *G. max*, around 15 MYA. This date is much older than that estimated by Delgado-Salinas et al.^43^ for the *Vigna* sensu lato crown clade (9.1 ± 1.0 MYA) from chloroplast *trnK* sequences. Protein evolution between orthologs was also assessed by calculating the Ka/Ks ratio, in this case, to identify patterns of selection after the speciation event separating *P. vulgaris* and *P. lunatus*. In line with previous studies in other species^38^, the distribution of Ka/Ks was centered far below 1, suggesting that most gene coding sequences evolve under purifying selection (Fig. 2b). Conversely, 656 gene pairs within the main synteny blocks showed rapid sequence divergence with Ka/Ks values larger than 1. Functional enrichment of these genes shows ontologies related to the metabolism of aminoglycan, chitin, and lignan (Supplementary Fig. 7). As described in the last section, genes related to these processes have increased expression values during the development of the pod.

### Orthologs of genes related to agronomic characteristics

Genes of agronomic interest were predicted by ortholog relationships of genes associated with agronomically relevant traits in other crops (Supplementary Data 1). We identified Lima bean orthologs of 30 genes having reported associations with traits in previous studies, 27 of which were reported in common bean. Traits included due to their importance in plant breeding are yield^44^, nutritional quality^45^, herbicide resistance^46^, plant architecture^47^, tolerance to abiotic stresses^48^, among others. Moreover, seed coat color and growth habit, important characteristics needed to meet consumer and farmer preferences in Lima bean and common bean breeding and marketing, were also included.

In particular, 1917 genes distributed across the 11 chromosomes were related to resistance to biotic stresses predicted on the base of bioinformatics analysis and the presence of the LRR (leucine-rich repeat-containing) and other important domains for disease resistance such as toll/interleukin-1 receptor (TIR), leucine zipper (LZ), coiled-coil (CC), nucleotide-binding site (NBS/NB) shared by ARC (Apaf-1, R proteins, and CED-4) (NBS/NB-ARC) domain, serine–threonine kinase, and WRKY (Fig. 2d and Supplementary Fig. 8). Serine–threonine and other receptor-like protein kinases were one of the most abundant types among the selected disease resistance candidate genes (828). The WRKY domain was present in 91 genes and the leucine zipper domain in 74. The CC domain was only found in 11 genes. However, a low number of CNL (CC-NBS-LRR) genes has been previously observed and reported in dicots^49^. In contrast, 98 TNLs (TIR-NB-LRR) were identified in the annotated gene models. Furthermore, 631 genes contained the LRR domain, 151 the NB-ARC domain, and 91 both domains (Supplementary Data 2). Large numbers of predicted disease resistance genes were localized to chromosomes Pl02, Pl04, Pl08, Pl10, and Pl11.

The subset of genes with the LRR domain tended to be clustered in discrete regions of the genome (Supplementary Fig. 9). This subset includes the following domain arrangements: LRR, NB-ARC-LRR, TIR-NB-ARC-LRR, and TIR-LRR. Neighbor joining clustering of this family showed some correspondence between chromosome clustering and sequence similarity (Fig. 2e). Proteins with the domains NB-ARC-LRR formed a large cluster, but five of them were nested into the TNL group that also contained the three proteins with the TIR and LRR domains only. Common bean orthologs were identified for most of the predicted genes related to biotic stress resistance. These orthologs were located on the same chromosomes and were mainly collinear. Also, motifs were mostly conserved between the gene sequences of both species. Furthermore, the genomic positions of the genes correspond with resistance loci associated by previous studies with some of the most important diseases affecting the common bean. For instance, we found clusters of genes within or close to resistance loci for anthracnose^50–54^, angular leaf spot^51,52,55^, halo blight^52,56^, bean golden yellow mosaic virus^51^; as well as other viral diseases, rust, and mildew^23,52^.

### Population structure analysis reveals genetic clusters in Lima bean

Wild Lima bean presumably originated in the northern Andes, during Pleistocene times, and from there it expanded to other areas in the Andes and Mesoamerica^11^. As a result, wild Lima bean reached a widespread distribution, from northern Mexico to northern Argentina, and became differentiated into three major gene pools (MI, MII, and AI) with mostly non-overlapping distributions, as documented by previous studies. Later, humans domesticated this species twice, once in Mesoamerica from gene pool MI and once in the Andes from gene pool AI^10,15,16^. To investigate in greater detail the genetic structure of Lima bean, we combined previously analyzed genotyping-by-sequencing (GBS) data from 270 Lima bean accessions^10^ with GBS data for 212 additional samples to increase the amount of variation (Supplementary Data 3). From a raw number of 116,030 biallelic SNVs, 12,398 were selected for diversity analysis (Supplementary Data 4).

**Fig. 3:**
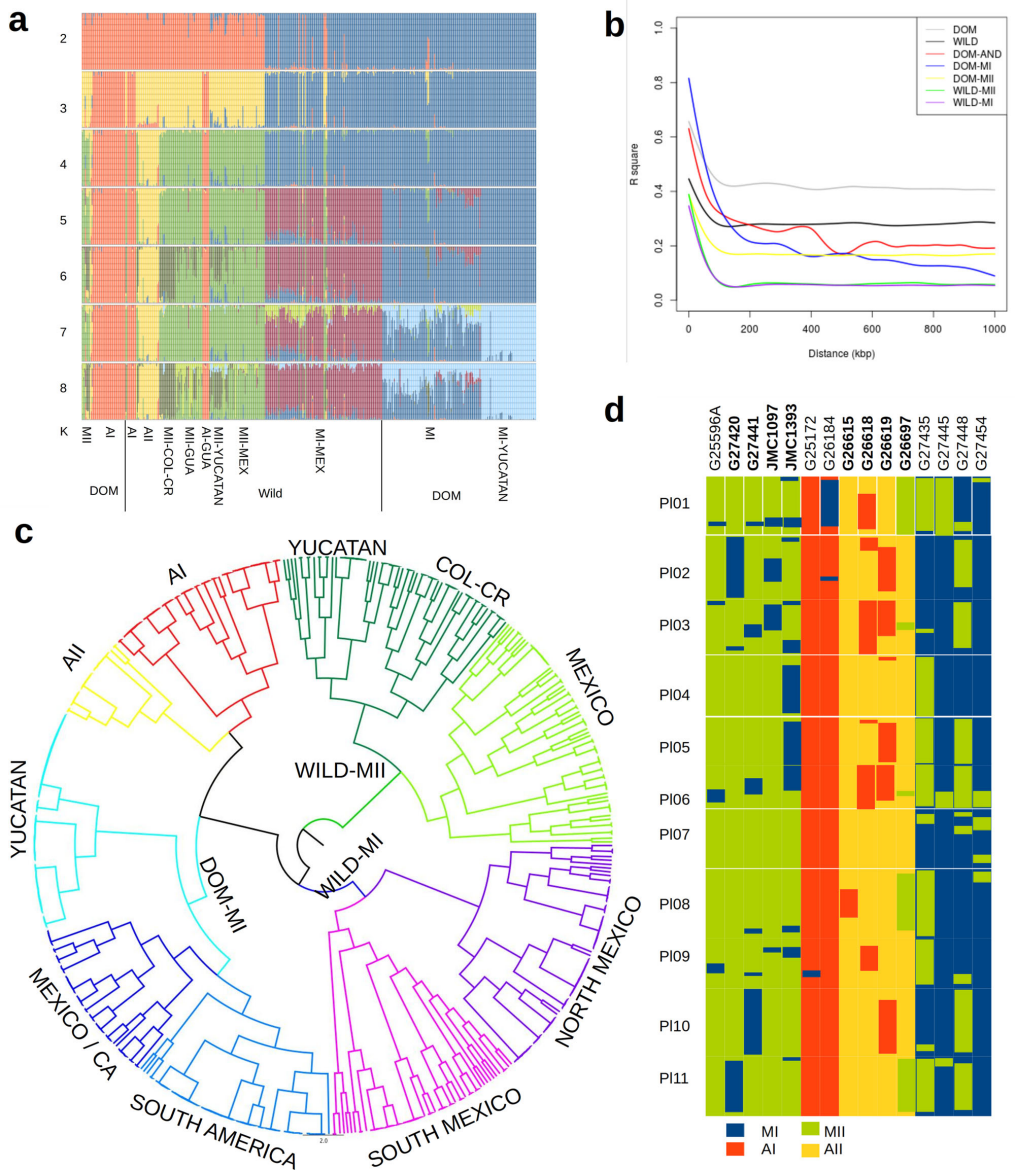
Lima bean genetic diversity. **a** STRUCTURE analysis of the genetic variability between 482 wild and domesticated Lima bean accessions collected across the Americas. Wild accessions are organized (from left to right) into a south-north geographic pattern. DOM domesticated, MEX Mexico, GUA Guatemala, CR Costa Rica, COL Colombia. Classification of accessions into gene pools MI, MII, AI, or AI is shown. **b** Linkage disequilibrium decay within different subgroups of wild and domesticated accessions. DOM domesticated, DOM-AND Andean landraces. **c** Radial clustering of the 482 accessions according to the analysis performed by fineSTRUCTURE. Major gene pools are shown by different colors (purple cluster: wild MI from northern-western Mexico (NORTH MEXICO); pink cluster: wild MI from southern-western Mexico (SOUTH MEXICO); medium blue cluster: domesticated MI from South America; dark blue cluster: domesticated MI from Mexico and Central America (MEXICO/CA); light blue cluster: domesticated MI from Yucatan Peninsula; yellow cluster: AII gene pool; red cluster: AI gene pool; green cluster: MII gene pool from Yucatan, Central America, and Colombia (YUCATAN COL-CR); light green cluster: MII gene pool from southern and central Mexico. **d** Distribution of chromosomal segments contributed by different gene pools in a set of 15 wild and domesticated accessions. Wild accessions are marked in bold. Lima bean chromosomes are labeled as Pl01-Pl11. Source data are provided as a Source Data file.

The samples sequenced in this study increased representation of the three major gene pools and the geographic sampling in countries such as the United States, Mexico, and Colombia (Supplementary Fig. 10). Different statistical and heuristic clustering analyses, including Neighbor Joining (NJ), discriminant analysis of principal components (DAPC), and Bayesian clustering (STRUCTURE) were applied. The optimal number of clusters according to the decrease of BIC is between *K* = 5 and *K* = 6, while STRUCTURE results suggest that the optimum *K* is 6 (Supplementary Fig. 11). The results not only supported the existence of the three major wild gene pools (MI, MII, and AI) but also showed evidence for two large novel clusters (Fig. 3a, Supplementary Data 5, and Supplementary Figs. 12–14). First, domesticated MI accessions were clearly separated from wild accessions in a single cluster, which is in agreement with a single domestication of Mesoamerican landraces (dark blue cluster in Fig. 3a from *K* = 5 onwards and Supplementary Fig. 12), and second, a more complete sampling of wild accessions in the central Andes of Colombia supported the presence and genetic differentiation of an Andean wild gene pool, the AII gene pool (yellow cluster in Fig. 2a, from *K* = 4 onwards and Supplementary Fig. 12). The wild AII group shows a very restricted distribution on the eastern slope of the Andes in central Colombia, specifically in the departments of Cundinamarca and Boyacá (although one accession occurs in Peru) (yellow dots in Supplementary Figs. 13, 14). It is worth noting that five wild accessions from Guatemala, one from Honduras, and one from Chiapas (Mexico) clustered within the Andean gene pool AI. When *K* = 6 is considered, a further subcluster is detected within wild gene pool MII (Fig. 3a. dark green cluster) that contains accessions from Peninsula of Yucatan in Mexico, northern Guatemala, Costa Rica, and northern Colombia.

Because Lima bean is an inbred species, heterozygosity rates per accession are generally low (details below), which facilitates the inference and analysis of haplotypes. Figure 3b shows the comparison of linkage disequilibrium (LD) decay between pairs of variants in the same chromosome. LD decays faster in wild accessions (dark gray line) than in domesticated accessions (light gray line). However, due to population structure, LD remains high (on average over 0.3 for the wild gene pool and over 0.4 for the domesticated gene pools) even at distances larger than 1 Mbp. LD analyses within wild and domesticated populations showed that in the wild MI and wild MII gene pools LD decays to basal levels at about 100 kbp, whereas in the domesticated MI, MII, and AI populations, it decays to background levels at about 500 kbp. LD decayed faster in MI and MII landraces than in the AI landraces, which agrees with the higher genetic diversity observed in the former (Supplementary Table 5).

Taking advantage of the reconstructed haplotypes and relatively long blocks of linkage disequilibrium, we applied the software fineSTRUCTURE^57^ to exploit the information contained in genome-wide linkage disequilibrium patterns for dissecting fine population structure. This approach not only identified a much larger number of populations than STRUCTURE (in total 181) but also revealed the genetic relationships among the inferred populations (Supplementary Data 5). Examination of the radial tree shown in Fig. 3c, from the highest to the lowest level of clustering, allowed to view the whole population at multiple structuring levels. In general, major clusters in the tree (shown by different colors) corresponded to the major gene pools detected by STRUCTURE, DAPC, and NJ. Also, the subgroups observed within the wild MI, Dom MI, and wild MII gene pools are clearly related to geography, as described below (Supplementary Figs. 13, 14).

Among the wild accessions, MI accessions were separated into two subgroups according to their geographical origin: one subgroup (purple cluster) included 55 accessions assigned to ten populations mainly distributed in northern-western Mexico, from the states of Sinaloa to Colima, and the other subgroup (pink cluster) included 34 accessions assigned to 13 populations mainly distributed in southern-western Mexico, from the state of Michoacan to Oaxaca. MII accessions were also separated into two subclusters: one of them (light green cluster) contained 66 accessions mainly distributed in Mexico and southern Guatemala, and the other one (dark green) contained 49 accessions from northern Guatemala, Costa Rica, northern Colombia, and nine accessions from the Peninsula of Yucatan. Among the domesticated accessions, MI landraces were separated into three subgroups according to their geographical origin: one subgroup (dark blue cluster) included 43 accessions assigned to 13 populations mainly distributed in Mexico and Central America, with only 12 accessions from other countries (United States, Colombia, and Ecuador), a second subgroup (medium blue cluster) included 51 accessions assigned to nine populations mainly distributed in South America, with only 8 accessions from the United States and one from Mexico, and the third subgroup (light blue cluster) contained 59 accessions assigned to five populations (numbers 19, 60, 80, 88, and 101. See Supplementary Data 5 and supplementary Fig. 15 for population numbers) distributed in the Yucatan Peninsula in Mexico. This third subgroup was also observed in the STRUCTURE results at *K* = 7 (Fig. 3a). By examining in more detail the landraces contained in each one of these five populations from the Yucatan Peninsula, there is some tendency to group accessions by variety or seed shape. According to the Mayan nomenclature registered by Martinez-Castillo et al.^58^, population 88 comprises 14 accessions that belong to four landrace varieties known as Bacalar-ib, Chak-saak, Chak-ib, and Bayo-ib, all of them with small and flattened or semi-flattened seeds (the typical morphotype of the Sieva cultigroup). Population 101 includes 24 accessions that belong to landrace varieties known as Putsica-Sutsuy, Mulicion blanco, Mulicion rojo, Box-ib, Pool-santo, Kan-ib, Morado-ib, Yete Boch ib, Kolbihi, and Chak ib, most of them characterized by having small rounded or semi-rounded seeds (the typical morphotype of the Potato cultigroup). Population 60 comprises 13 accessions that belong to varieties known as Sac-ib, Chak-chi, Bayo-ib, Mejen-ib, Bacalar, and Madza-kitam with seed morphology intermediate between both cultigroups. Population 80 contains four landrace accessions that belong to the variety known as Box-ib that carry small purple-black semi-flattened or rounded seeds. Population 19 contains 4 accessions of the landrace variety known as Chak-chi with white-red small seeds with seed morphology also intermediate. The intermediate forms may arise by the fact that some farmers in the Yucatan Peninsula may grow up to seven different landrace varieties together, which may promote opportunities for crossing^58,59^.

Supplementary Table 5 shows basic diversity statistics for all major gene pools in wild and domesticated accessions. Observed heterozygosity was much lower than expected heterozygosity, as expected for an inbred species as Lima bean (Bartlett’s K-squared = 18257, df = 1, *p* value < 2.2e-16; paired-t test *t* = 165.28, df = 12397, *p* value < 2.2e-16). Among the wild clusters, the most diverse were the Mesoamerican MI and MII gene pools (*H*_E_ = 0.128 and *H*_E_ = 0.133, respectively), while the least diverse were the Andean AI (*H*_E_ = 0.040) and AII gene pool (*H*_E_ = 0.052). The genetic diversity of the seven wild accessions from Chiapas, Guatemala, and Honduras, clustered within the AI gene pool, was also low (*H*_E_ = 0.053). Domestication brought a reduction in genetic diversity in landraces (the domestication founder effect). When measured in the whole sample, this reduction was about 25%, but when measured within major gene pools, the reduction was more drastic for the Mesoamerican domestication (MI gene pool, 55%) than for the Andean domestication, where no reduction was observed. It is worth noting that the genetic diversity of MI landraces from the Yucatan Peninsula (*H*_E_ = 0.029) is significantly lower than other MI landraces (*H*_E_ = 0.065). This result is in agreement with a late introduction of the crop in the Yucatan Peninsula from its area of origin in central-western Mexico, as well as a late development (or introduction) of agriculture in the Maya Lowlands^60^.

Pairwise *F*_ST_ distances among gene pools showed that the wild gene pool most closely related to the Mesoamerican landraces is MI (*F*_ST_ = 0.33), distributed in central-western Mexico (Supplementary Table 6). As for the Andean landraces, the most closely related wild gene pool is AI (*F*_ST_ = 0.21), mostly distributed in the Andes of Ecuador-northern Peru. *F*_ST_ values also showed that the wild cluster, AII is most closely related to the Mesoamerican gene pools (*F*_ST_ values ranged from 0.56 to 0.65) than to the Andean gene pool AI (*F*_ST_ = 0.86). Such a close relationship was also shown by Caicedo et al.^61^ on the basis of AFLP polymorphisms and Toro et al.^62^ on the basis of electrophoretic patterns of phaseolin. STRUCTURE results also suggest a close relationship of gene pool AII with gene pool MII (Fig. 3a), however, the analysis of fineSTRUCTURE shows that gene pool AII is most closely related to gene pool AI (Fig. 3c).

As stated above, *F*_ST_ values showed high genetic differentiation among gene pools, and consistent with this, haplotype introgression analyses clustered most of the accessions within their respective gene pools. However, there were 103 instances of chromosomal segments distributed in 35 accessions (9 wild and 26 domesticated) that could represent genetic contributions from different gene pools (Supplementary Data 6). The 103 chromosomal segments varied in size from 1 to 53 Mbp. In six accessions these chromosomal segments occupy more than 25% of their genome length, and concordantly these accessions were classified by STRUCTURE as admixed. Figure 3d shows the 58 chromosomal segments that were larger than 5 Mbp and that were observed in 15 accessions. Most of these segments represent genetic contributions between Mesoamerican gene pools (MI and MII) or between Andean gene pools (AI and AII), and more rarely between Mesoamerican and Andean gene pools.

These genetic contributions may be the result of recent contact between wild and domesticated accessions, or between domesticated accessions of different origin. We found examples where introduced domesticated populations may have contributed chromosomal segments to the genetic makeup of wild accessions. For example, five MII wild accessions, distributed in the Mexican states of Chiapas and Campeche, in Guatemala, and in northern Colombia, all carry chromosomal segments that could have been potentially introgressed from introduced MI domesticated accessions. In all these geographical places, wild MII and MI domesticated accessions are distributed. Two AII wild accessions from central Colombia carry chromosomal segments potentially derived from domesticated AI accessions. We also observed 18 MI domesticated accessions carrying chromosomal segments potentially derived from the MII gene pool. These could represent cases of introgression from MII wild accessions into MI landraces in places where both kinds of accessions coexist such as the Mexican states of Veracruz and Chiapas, Costa Rica, El Salvador, and the Caribbean coast in northern Colombia. Interestingly, we detected four MI landraces from the United States and one from Colombia with chromosomal segments potentially derived from Andean AI landraces. At least in Colombia and Ecuador MI and AI landraces coexist. We also found two AI landraces, G25172 and G26184 collected in Ica, Peru, where the foreign haplotypes belong to the MI gene pool suggesting that these accessions might be the result of an interbreeding occurrence, maybe due to the presence of both Andean and Mesoamerican landraces in Ica, Peru. An early introduction of Mesoamerican landraces in coastal Peru has been suggested by the occurrence of pod remains (typical of small-seeded landraces) in association with the ceramics of the Guanape Period (from 1200 B.C. to 400 B.C.) and Cupisnique Period (from 1500 B.C. to 500 B.C.) in Huaca Prieta, Peru^63^, although more recent introductions cannot be excluded. Finally, an interesting pattern that we observed was that a single 2.6 Mbp MII segment located in chromosome Pl07 was shared by six MI landraces from different departments in Colombia, and that a second 2.2 Mbp MII segment located in chromosome Pl08 was shared by seven different MI landraces cultivated in different places of Central America and Colombia (Supplementary Data 6).

### Gene expression during pod development

**Fig. 4:**
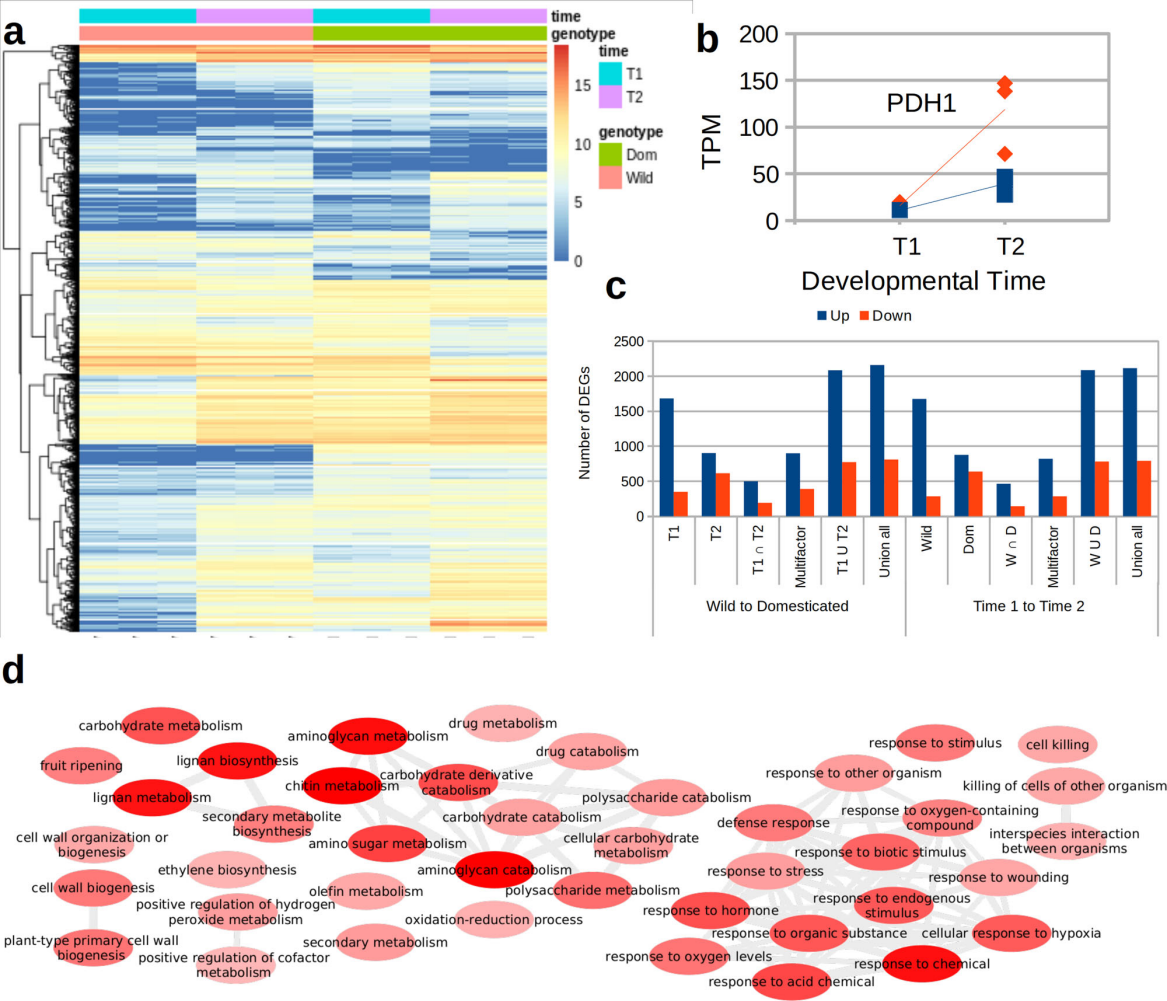
Gene expression at pod developmental stages T1 and T2. **a** Heatmap of normalized expression values within genes with differential expression. The left dendrogram corresponds to an unsupervised hierarchical clustering of the genes based on the normalized expression values. **b** Expression trajectories of the gene *PlPDH1* in the domesticated accession (blue) and the wild accession (red) across two developmental times. TPM, transcripts per million. **c** Number of genes with differential expression (DEGs) between one wild and one domesticated accession and between two developmental times. **d** Concept map of functional categories enriched for genes more expressed in the second developmental time only in the wild accession. Source data are provided as a Source Data file.

Reduction or loss of pod dehiscence is one of the key domestication traits in Lima bean, and also a trait of agronomic importance^64,65^. To obtain information on the genetic regulation of the pod development, we carried out an RNA-seq experiment measuring expression levels at the initiation of pod elongation (T1) and before seed filling (T2) in one wild and one domesticated accession. At the developmental stage T1, pod valves become visible with the flower corolla still attached (or recently detached). At the developmental stage T2 pods reach their maximum length and weight (excluding the seeds) before the initiation of seed filling. Principal component analysis of expression values inferred from RNA-seq reads, including publicly available reads from a previous study^66^ consistently clustered replicates of each library with only one outlier which was removed for downstream analysis (Supplementary Fig. 16). Differential expression (DE) analyses revealed a total of 4275 genes with patterns of differential expression either across developmental stages or between the wild and the domesticated accession (Supplementary Data 7). Figure 4a shows a general heatmap of normalized expression values for these genes. Hierarchical clustering based on these values distinguished between five and seven gene clusters following different expression patterns.

Looking at genes previously identified as related to pod dehiscence, the *PDH1* gene significantly increased expression between T1 and T2 (Fig. 4b). The expression at the second stage was over 2-fold higher in the wild accession compared to the domesticated accession but the difference between accessions was not significant due to a relatively low expression value of one of the replicates. The *PDH1* gene is involved in the formation of fibrous, strongly lignified cell layers between the inner and outer parenchyma of the pod, thus increasing the torsion force of pods in shattering genotypes^67^. Furthermore, it has been recently identified as a strong pod dehiscence QTL on chromosome Pv03 in common bean^65^.

The ratio of gene expression between developmental stages or accessions showed that in general the domesticated accession had increased expression values for a larger number of genes than the wild accession (especially at T1) and that the number of genes with increased expression is larger than the number of genes with decreased expression between T1 and T2, especially in the wild accession (Fig. 4c). These patterns are collectively explained by about 1500 genes increasing expression between T1 and T2 in the wild accession. Genes with consistently increased expression between T1 and T2 in both the wild and the domesticated accession are enriched for terms that include cell wall biogenesis and organization, which is related to the biosynthesis of polysaccharides, particularly xylan and lignin, important components of the seed (Supplementary Fig. 17). Conversely, genes that only increase expression between T1 and T2 in the wild accession are enriched for metabolism-related genes of lignan, chitin, and aminoglycan and the fruit ripening process (Fig. 4d). Enrichment of chitin metabolism-related genes was mainly generated by a cluster of seven genes located at 38 Mbp of chromosome Pl09. Lignan metabolism-related genes were also enriched in this subset, mainly due to an array of nine genes with increased expression located at 9 Mbp of Pl11. Genes that remained more expressed in the domesticated accession only at T2 show enrichment of functions related to the development of the reproductive system, and particularly with the development of the fruit (Supplementary Fig. 18). Finally, genes with consistently reduced expression between T1 and T2 were enriched for developmental processes such as cuticle development and metabolism of different compounds, including cyanogenic glycoside. These findings are expected because the plant completed the formation of the pod and is about to start filling the seed.

## Discussion

In this work, we conducted a large collaborative effort to provide a comprehensive set of genetic and genomic information for Lima bean. This effort resulted in information on the content, organization, and function of chromosomes and sequences, evolutionary relationships with close relatives at different taxonomic levels, population genomics of wild and cultivated accessions, and inheritance of agronomic traits. The analysis of this information revealed, at a greater detail, the genetic structure of wild and domesticated Lima beans across their large distribution range in the Americas, and provided insights on the genetic basis of variability in different agronomically relevant traits. Knowing the genes controlling these traits represents a great advantage for breeding programs and could potentially accelerate the development and release of improved Lima bean varieties in the future.

The backbone of these achievements is the chromosome-level genome assembly for *P. lunatus* and a comprehensive genotyping of the available genetic variability within the species. The effort to build a high-quality reference genome sequence, both in terms of contiguity and base-pair quality, was rewarded by the assembly and functional prediction of large clusters of genes related to different traits. Although these predictions must be experimentally validated, the information provided is useful to prioritize genes to perform experimental validation as a community effort. Genes that confer resistance to biotic stresses on plants show large nucleotide diversity but good correspondence with common bean resistance genes based on orthology predictions and literature. As observed in previous studies in species with high-quality genomes such as rice, the variability of disease resistance genes is a key component of the defense mechanisms in plants^68^ and can contribute to functional redundancy favoring durability of resistance in the field^69^. Moreover, local gene duplication was also observed in genes performing functions such as metabolism of xyloglucan and chitin and regulation of flower development, which are directly related to yield traits such as seed weight and flowering time.

The evolutionary history of plant genomes is shaped by several WGD and local duplication events, characterized by a wide range of evolutionary rates within and between gene families with important consequences in gene expression and function^38^. A full understanding of these processes is only possible through the development of high-quality genome sequences^29^. In the case of *Phaseolus* species, a synteny analysis of our assembly with that of *P. vulgaris* not only confirms the high degree of chromosome conservation between these genomes but also provides a detailed view of five major rearrangements between these genomes. Thus, this work makes a significant contribution to ongoing genome assembly and resequencing efforts to allow a full reconstruction of the evolution of genomes within the legume family, including the complete characterization of potential convergent evolution processes triggered by multiple domestication events.

Previous phylogenetic analyses have placed the origin of wild Lima bean in the Andes of Ecuador-northern Peru during Pleistocene times and have suggested that, in this ancestral area (gene pool AI), wild populations experienced a range fragmentation event that reduced genetic diversity^11^. Accordingly, we found that Andean wild populations are relatively less diverse than the Mesoamerican ones, although more sampling is needed to have a more precise estimation of genetic diversity in this population. In wild common beans, a reduction of diversity was also documented in Andean populations but it was attributed to the effect of rare long-distance dispersal events from Mesoamerican populations to the Andes^70^. The identification of a wild Lima bean cluster (AII) in the Andes of central Colombia is of great significance because it supports the dispersal of the species from south to north and may represent a source of alleles not found elsewhere, as suggested by the characteristic phaseolin type (M8) that has been observed in this gene pool (see Supplementary Data 3). With regard to domestication, this study revealed that the domestication bottleneck in Mesoamerica seems to be more severe than previously thought^10^. The genetic diversity that was lost during domestication was apparently hardly re-gained by gene flow from wild to domesticated types, as suggested by the low number of landrace accessions showing signals of introgression. However, further studies including whole genome resequencing of larger numbers of landraces in a wider range of geographic locations are needed to completely assess the role of introgression in the current genetic diversity of domesticated Lima bean and avoid potential biases produced by low sampling or missing data in GBS experiments.

The higher number of samples and genetic markers analyzed in the present study also provided us more in-depth information on the genetic substructure of the MI gene pool and its relation to geography, although we acknowledge that there could still be some sources of bias due to the relatively low SNP density and missing data obtained from GBS experiments. Within the MI wild cluster, two subclusters were observed: northern-western Mexico and southern-western Mexico. Also, within the cluster of Mesoamerican MI landraces, three subclusters were detected: Mexico-Central America, South America, and the Peninsula of Yucatan. The observation that landraces from the Peninsula of Yucatan form a separate subcluster and that their genetic diversity is relatively lower than other MI landraces, is in agreement with the hypothesis that after its domestication in central-western Mexico, Lima bean was a relatively late adoption of the Mayan communities of the Peninsula of Yucatan^60^. Unfortunately, in this region, Lima bean is under serious threat of genetic erosion^58,71^. The genetic differentiation observed among wild gene pools, the predicted domestication bottleneck in Mesoamerica and the threat of genetic erosion of landraces in the Yucatan Peninsula have important implications for conservation purposes.

The development of the biparental population UC Haskell–UC 92 presented in this work not only guided the genome assembly but also provided a unique tool for further investigation on the drivers of domestication traits through QTL mapping. As in common bean^72^, the presence of the *PlTFL1* gene is associated with a QTL for both determinacy and flowering time: determinacy can cause earlier flowering by converting the terminal meristems from a vegetative state into reproductive ones. In common bean, the determinacy trait is more common in the Andean gene pool^73^. In this experiment, the determinacy trait was contributed by the Andean parent UC 92. This observation raises several questions. Given the presence of determinacy in the Mesoamerican gene pool as well, what is the origin of this trait in the latter gene pool? Is it due to an independent mutation at the same or different loci or is due to introgression from the Andean gene pool? Whole genome resequencing data among a broader sample of Andean and Mesoamerican accessions is necessary to answer these and other questions.

The three quantitative traits scored in the UC 92–UC Haskell population showed transgressive segregation to varying extents. However, seed weight only showed transgressive segregation for seed weights below the small-seeded parent UC Haskell, but not for larger seed weights. This observation points to the difficulty in developing large-seeded improved progenies in populations arising from selfing only. Possible solutions are the development of population based on backcrosses to a large-seeded parent. An alternative is suggested by the phenotypic distribution for HSW, discriminated by allelic combinations (Supplementary Fig. 6). The presence of three marker alleles of the large-seeded parent (UC 92) for three seed weight QTL corresponds to the heaviest seeds and vice-versa for the individuals with the three marker alleles of the small-seeded parent (UC Haskell). Thus, indirect selection using these markers in early generations may shift breeding populations towards heavier seeds. Regarding cyanogenesis, the combination of QTL alleles giving the highest levels of cyanogenic glucosidase was the Pl05 (UC Haskell)–Pl08 (UC 92)–Pl10 (UC Haskell). Whether or not this combination should be selected for will depend on considerations such as a concern for consumer safety or a potential role in insect resistance.

We also used RNA-seq to follow the gene expression patterns at two pod developmental stages in one wild (shattering) and one domesticated (non shattering) Lima bean accession. Hundreds of differentially expressed genes between the two accessions were detected between two developmental stages of the pod (T1 at the initiation of pod development and T2 when pods reached their maximum length before seed filling). In particular, we identified differential expression in the *PDH1* gene, known to be involved in the control of pod dehiscence in other crops^74^. The *PDH1* gene is a ‘dirigent’-type gene involved in the polymerization of lignin monomers, consistent with the pod fiber role in dehiscent pods of wild types. In soybean, this gene is involved not only in lignification of pod walls but also in the increase in the torsion force of the pod^67^. Hence, the reduced expression of this gene is consistent with reduced pod dehiscence in domesticated types.

In *P. vulgaris*, *PvPDH1* is known to be only transcribed in the pod tissue^75^. Recently, this gene has been associated with pod dehiscence and with the adaptation of the domesticated ecogeographic race Durango of *P. vulgaris* to arid conditions in northern Mexico^65^. Anatomical and histological analyses have shown that shattering genotypes have an extensive lignified wall fiber layer (LFL) in pod walls while in non-shattering genotypes LFL deposition is reduced or absent. Our observation that the *PlPDH1* gene increases expression in the wild accession of Lima bean at T2 is compatible with the results in common bean; therefore, *PlPDH1* is a good candidate for further investigation. Moreover, the information obtained from differential expression was also useful to identify genes related to the regulation of flower development and metabolism of xylan and pectin, which in turn can be related to flowering time and seed weight. In addition, interesting patterns of expression were also observed for genes with annotated biological processes such as metabolism of chitin and cyanogenic glycoside, cuticle development, response to auxin, cell wall biogenesis and fruit ripening, which could be related to different physiological characteristics and even seed quality traits such as cookability or cyanide content.

Given the importance of Lima bean, both as a current food security crop and as a potential protein source for different climate change scenarios, we expect that this work will provide a basis for many future studies in *Phaseolus* species with applications to breeding and will make an important contribution to the field of *Phaseolus* genomics across the five domesticated species of the genus.

## Methods

### Sequencing

Lima bean genomic DNA was extracted from young leaves of two-week-old seedlings of the domesticated accession G27455 according to the requirements for DNA concentration and integrity of each sequencing technology. High molecular weight (HMW) DNA for Pacific Bioscience and 10X Genomics sequencing was extracted with the Qiagen MagAttract HMW DNA Kit (QIAGEN, Germantown, MD, USA) following manufacturers’ instructions. For the construction of libraries for Pacific Bioscience sequencing, a SMRTbell Template Prep Kit was used according to the manufacturer’s instructions (Pacific Biosciences, Menlo Park, CA, USA). DNA was randomly fragmented by adding fragmentation buffer using Covaris g-TUBE devices and the purification was carried out with AMPurePB magnetic beads after concentration. Fragments greater than 3 kbp underwent a damage repair step combined with an end-repair, followed by a blunt end ligation with hairpin adapters. Libraries were sequenced on a PacBio Sequel platform at the sequencing provider Novogene Corporation Inc.

For 10X Genomics DNA library construction, one μg of Lima bean DNA was prepared with Chromium Genome HT Library & Gel Bead Kit V2/10x Genomics according to the manufacturer’s protocol (10x Genomics, Pleasanton, CA, USA). The resulting 500–700 bp insert libraries were quantified using a Qubit 2.0 fluorometer (Thermo Fisher Scientific, Waltham, MA, USA) and quantitative PCR. The size distribution was analyzed using an Agilent 2100 Bioanalyzer (Agilent Technologies, Santa Clara, CA, USA). Suitable libraries were sequenced on an Illumina HiSeq Platform (Illumina, San Diego, CA, USA) using a paired-end 150 run (2 × 150 bases).

For Illumina sequencing, young trifoliate leaves from two-week-old seedlings were collected and frozen with liquid nitrogen. DNA isolation was performed with the same extraction method used for genetic diversity and population structure analyses (see below). The Illumina library used 1.0 μg of DNA according to a NEBNext DNA Library Prep Kit following the manufacturer’s recommendations (New England BioLabs, Ipswich, MA, USA). Genomic DNA was fragmented to a size of 350 bp, fragments were ligated to NEBNext adapters and enriched by PCR. The library was analyzed for size distribution with an Agilent 2100 Bioanalyzer (Agilent Technologies, Santa Clara, CA, USA) and quantified using real-time PCR. Libraries were sequenced on an Illumina HiSeq Platform (Illumina, San Diego, CA, USA) using a paired-end 150 run (2 × 150 bases) and insert size of 450 bp.

### RNA sequencing and de novo transcriptome assembly

Plants from accessions G25230 (Mesoamerican wild) and G27455 (Mesoamerican landrace) were grown under greenhouse conditions at the Centro Internacional de Agricultura Tropical (CIAT; Palmira, Valle del Cauca, Colombia). Total RNA was extracted from leaves, pods, and flowers with a specific protocol for each tissue^76,77^. For the analysis of differential gene expression related to pod dehiscence, RNA was extracted for the wild and domesticated accession at two pod developmental stages (with three replicates each), at the initiation of pod elongation (T1) and before seed filling (T2). At T1 pod valves become visible with the flower corolla still attached (or recently detached) and at T2 pods reach their maximum length and weight before seed filling. Previous studies show that strong lignin deposition at the dehiscence zone is already observed at this stage of the pod development in wild samples^78^. RNA samples were quantified with Nanodrop 2000 and quality was assessed with Agilent Bioanalyzer 2100 system (Agilent Technologies, Santa Clara, CA, USA) and agarose gel electrophoresis. Samples showing a 260/280 ratio of absorbance between 1.9–2.0 and RIN (RNA Integrity Number) values above seven were selected. mRNA samples were enriched using oligo(dT) beads and randomly fragmented, then cDNA was synthesized using mRNA templates. RNA-seq library preparation was completed through size selection and PCR enrichment. Sequencing of the library was accomplished using a paired-end 150 run (2 × 150 bases) on an Illumina HiSeq Platform (Illumina, San Diego, CA, USA).

The quality of the raw reads was evaluated with the fastQC v.0.11.2 program^79^ and low-quality reads and adapter sequences were filtered with Trimmomatic v.0.36^80^, removing approximately 3% of the reads. De novo transcriptome assemblies were obtained by using the software Trinity v.2.4.0^81^. Each transcriptome assembly was compared with 1440 single-copy orthologs from the OrthoDB v.9.1 database using the BUSCO (Benchmarking Universal Single-Copy Orthologs) v.2 pipeline^82^.

To increase the expression evidence for gene annotation, we also downloaded public RNA-seq reads sequenced in a previous study^66^ from the NCBI Sequence read archive (bioproject accession number PRJNA275266), using the fastq-dump facility of the SRA toolkit v.2.9.4 (https://github.com/ncbi/sra-tools/wiki).

### Sequencing and assembly into scaffolds and pseudomolecules

A chromosome-level assembly of the Lima bean genome was achieved by combining reads from four different sequencing protocols (Supplementary Table 7). The entire sequencing effort added up to a total of 97.6 Gbp of raw data, which represents about 157x of the haploid genome size, initially estimated to be around 622 Mbp^27^. The genome assembly pipeline included four main steps. First, a de novo assembly of the PacBio reads was performed using Canu v.1.6 with default parameters^83^. This step resulted in a draft assembly of 496 contigs with N50 of 5.5 Mbp and a total length of 542 Mbp (Supplementary Fig. 19). An initial analysis of the linked reads data and the GBS data from the biparental population (see details below) allowed us to identify and break 12 potential misassemblies. As a second step, polishing was performed to achieve high base pair quality integrating the paired-end Illumina data to correct base pair and indel errors. Reads were aligned to the 508 contigs using bowtie2 v.2.3.5^84^ and variants were called using the command FindVariants of NGSEP 3.3.2^85^ with the following parameters: -runRep, -runRD, -runRP, -minMQ 0, -maxBaseQS 30, -minQuality 40, -h 0.0001 and providing predictions of STRs performed using tandem repeats finder^86^. Assembly errors, called as homozygous alternative variants, were corrected using the command VCFIndividualGenomeBuilder of NGSEP. This process was repeated four times. The options -runRep and -runRD were executed to identify repetitive regions from multiple alignments and CNVs from read depth signal, respectively. Although 439 contigs (86%) were identified as repetitive because they included predicted repeats over at least 60% of their total length, the total length spanned by these contigs is 194 Mbp (36% of the assembled length). The remaining 69 scaffolds span 348 Mbp mostly because they are the longest contigs (Supplementary Fig. 20). In a third step, linked reads were aligned to the polished contigs using bwa v.0.7.17^87^ to perform scaffolding. In the 10x protocol, reads having the same barcode should have been sequenced from the same initial molecule. Hence, reads with the same barcode mapping to different contig ends provide evidence of linkage between such ends. We built an undirected graph having contig ends as vertices and evidence of linkage as edges to run a clustering algorithm similar to that implemented in the software Salsa for Hi-C data^88^. This procedure allowed us to combine 164 contigs in 40 scaffolds.

Finally, in a fourth step, a dense genetic map including more than ten thousand selected variants was built by analyzing GBS data from an F_8_ biparental population (parents UC 92 and UC Haskell, details in the next section). Linkage disequilibrium was calculated for each pair of variants and pairs in different contigs having an r^2^ value greater than 0.8 were considered as evidence for linkage between contigs. Connected components on a graph with contigs as vertices and linkage evidence as edges allowed us to identify the expected 11 linkage groups. Combining this linkage information with the linkage provided by the scaffolding process, 206 contigs adding up to 512 Mbp were sorted and oriented to produce the final 11 pseudomolecules representing the chromosomes of the species. The GBS reads were reanalyzed against the assembled pseudomolecules to verify the structural consistency of the final assembly and to identify recombination breakpoints per sample.

### RNA-seq data-based genome annotation, comparisons, and expression analysis

Annotation of repetitive elements was performed using RepeatMasker v.4.0.5^30^ using the library of repetitive elements for *P. vulgaris* kindly provided by the authors of Gao et al.^31^. The genome assembly was masked by replacing nucleotides within these regions with N characters to perform gene annotation. The genome annotation strategy combined ab initio predictions, cDNA sequences (pod, flower, and leaves), and homology-based approaches following the Maker pipeline v.2.31.9^89^. Raw RNA-Seq data were filtered using Trimmomatic v.0.36 to remove adapter and low-quality sequences (>Q30). We used 57,742 de-novo transcript assemblies obtained following the Trinity pipeline^81^ and 36,995 putative protein sequences from common bean as evidence in the Maker annotation process. In parallel, RNA-seq clean reads were processed according to the Tuxedo pipeline^90^. HISAT2 v.2.1.0^91^ was used to align the reads to the Lima bean reference genome with default parameters. StringTie v.1.3.5^92^ was used to predict transcript annotations from aligned reads. The two annotations were merged using a custom script available with the NGSEP distribution (class ngsep.transcriptome.io.GFF3CombineAnnotations). Suspiciously long genes and genes with an abnormally high number and distribution of transcripts and covering other genes were curated according to sequence similarity with common bean. This annotation was initially used for validation of the base pair quality of the genome assembly searching 1440 single-copy orthologs from the OrthoDB v9.1 database using the BUSCO (Benchmarking Universal Single-Copy Orthologs) v.2 pipeline^82^. The Tuxedo pipeline was also followed to generate the count of expression levels for each gene and each sample. HISAT2 v.2.1.0 was used to align the reads to the Lima bean reference genome and StringTie v.1.3.5 was used to obtain the matrix of read counts. We carried out a differential expression analysis with DeSeq2 v.3.1^93^ performing independent comparisons between developmental stages for each accession and between accessions within each developmental stage. Results of a linear model design combining developmental stages and accessions were also evaluated. Significant differential expression was predicted if the comparison *p* value was below 0.05 and the log-fold change was above two. To assess at which level the results were skewed by reference bias, expression levels for the transcripts assembled de-novo were estimated directly from raw reads using the software tool Salmon v.1.2.1^94^. Differential expression analysis of these expression levels was also assessed with DeSeq2. The trends were consistent between expression levels obtained following the two pipelines (Supplementary Fig. 21).

Functional annotation of the predicted gene models and transcripts was performed following the Trinotate pipeline v.3.1.1^95^. In brief, blastx and blastp searches from NCBI Blast v.2.10.0 were performed against the UniProt database using as queries the cDNA and amino acid sequences of each transcript, respectively. The best hit of each search was retrieved if its corresponding *e* value was below 0.001. The hmmscan tool of HMMer v.3.3.1^96^ was also used to identify conserved domains registered in the Pfam database. Results of these queries were combined into a Trinotate database to generate the final annotation report.

To assess the quality of the gene models and perform automated curation, orthology with *P. vulgaris*, expression measured as having a transcript per million (TPM) measure greater than 0.5 in at least one tissue and functional annotation were considered evidence supporting the existence of each annotated gene. A total of 10,263 annotations were filtered keeping genes with at least one type of evidence or having both proteins length over 200 amino acids and a paralog with direct evidence.

Paralog identification and comparison with other annotated genomes were performed running the GenomesAligner command of NGSEP. In brief, this command builds an FMIndex with the proteomes of each species and then performs amino acid searches of non-overlapping k-mers taken from each sequence on each FM-Index. A homology relationship is called if the percentage of matching query k-mers for a sequence is larger than a given parameter. To achieve identification of paralog relationships from the latest whole-genome duplication event, the GenomesAligner was executed with a k-mer length *k* = 5 and a minimum percentage of k-mers *p* = 20. A WGD paralog relationship is called if the two genes have at most ten paralogs in total, are located in different chromosomes and at least half of the neighbors in a window of up to ten genes share a syntenic homology relationship.

Functional enrichment of gene ontology (GO) terms for gene sets selected from the different experiments was performed running the topGO package of Bioconductor v.2.36.0^97^ using a Fisher exact test. Each set was compared against the genes in the Lima bean genome as background. Visualization of enriched GO terms was performed using REVIGO^98^ and visualization of GO knowledge graphs was performed in Cytoscape v.3.8.1^99^.

Identification of genes for biotic disease resistance was performed based on the functional annotation of the genome and orthology prediction. The complete list of gene models with known functions was manually screened for known domains associated with resistance (*R*)-genes as predicted or confirmed by at least one of the searched databases: NCBI (BLASTp), Pfam, and eggNOG/GO/KEGG. The domains included in the selection were: CC, NB-ARC, TIR, LRR, WRKY, LZ, and protein kinase. According to the different domain arrangements within the nucleotide-binding site (NBS)-Leucine-rich repeat (LRR) gene family different subfamilies were classified for each of the 11 Lima bean chromosomes. Genomic locations of these genes and their different protein sequences were derived from the genome assembly and structural annotation. A physical map of the 11 chromosomes with individual NBS-LRR genes was generated by MapGene2Chrom web v.2.1^100^ and a multiple sequence alignment of the corresponding proteins was created using muscle v.3.8.31^101^. The alignment was used to produce a tree topology for gene diversity analysis based on the Neighbor-joining approach by MEGA X v.10.8.1^102^, which was visualized and edited by iTOL v.4.4.2^103^.

### Genetic analyses in the UC 92 and UC Haskell recombinant inbred population

A biparental recombinant inbred line (RIL) population was developed at the University of California, Davis (38.542790° N. Lat.; 121.763049° W. Long.) from reciprocal crosses of two contrasting California Central Valley-adapted cultivars, UC 92 and UC Haskell, from distinct domestication gene pools of Lima bean. UC Haskell is a small-seeded, vine-type cultivar of Mesoamerican origin and UC 92 is a large-seeded, bush-type cultivar of Andean origin. Crosses were made in a greenhouse in 2012 and progeny seeds were grown for hybrid verification. F_1_ hybrids resulting from this cross were verified using the polymorphic codominant Pvctt001 simple sequence repeat (SSR) marker using a PCR protocol performed as recommended using Taq DNA Polymerase with ThermoPol Buffer (New England Biolabs). PCR products were analyzed on a 2% agarose gel using 1x TAE buffer. Subsequently, the population was advanced to the F_8_ stage by single-seed descent. Leaf tissue of the two parents and each RIL was collected from two-week-old seedlings; DNA was extracted using a DNEasy Plant Mini Kit (Qiagen). The resulting RIL population demonstrated segregation for many agronomic traits, including germination rate, flowering time, inflorescence position, plant height, plant habit, pod position, pod density, yield, and biotic stress tolerances^21^.

Leaf tissue for DNA extraction was sampled from two-week-old seedlings of 238 RILs in the F_8_ generation. Leaf tissue samples were collected into 96-well plates from plants grown in a greenhouse, immediately put on ice, and lyophilized for 24 h. DNA was extracted using an adapted DNA extraction protocol for Lima and common bean^104^. The presence of DNA was confirmed for samples with 260/280 absorbance ratios above 1.8 using a NanoDrop Lite (Thermo Fisher Scientific). DNA was quantified using the Quant-iT PicoGreen dsDNA Assay Kit (Thermo Fisher Scientific) and 100 ng of DNA from each sample was transferred to a PCR plate. GBS barcode libraries and adapters for the *Cvi*AII restriction enzyme (New England Biolabs) were prepared using an adapted protocol for common bean^104^. The *Cvi*AII restriction enzyme^104^, CutSmart buffer (New England Biolabs), and unique barcode identifiers for each well were added to the plates, spun down and incubated in a PCR machine for 2 h at 25 °C. T4 (10x) buffer and T4 ligation were added to the wells and run in the PCR machine for 1 h at 22 °C followed by 30 min at 65 °C. Seven microliters of each sample was added to a petri dish, mixed, and transferred to Eppendorf tubes. Binding buffer and isopropanol were added to the tubes and mixed. Eight-hundred microliters were transferred to a GeneJET (Thermo Fisher Scientific) purification column, centrifuged for 60 s, and eluted with water. DNA was quantified on the QUBIT dsDNA HS Assay Kit (Thermo Fisher Scientific/Invitrogen) prior to GBS sequencing. Two genomic libraries were prepared, each containing 144 unique barcode identifiers for the *Cvi*AII restriction enzyme, and a total of 240 unique genetic lines were sequenced. The libraries were sequenced using the SR100 protocol on two lanes of an Illumina HiSeq flow cell at the University of California, Davis, Genome Center.

Sequence data was de-multiplexed and adapter contamination was removed using the Demultiplex command of NGSEP. Single reads for each sample were aligned to the contigs using bowtie2 with default parameters. Then, the command MultisampleVariantsDetector of NGSEP was used to identify variants and call genotypes for each sample with parameters similar to those used to analyze the GBS diversity data. A total of 51,897 SNVs, 2927 biallelic indels, and 758 biallelic STRs were identified in 183 contigs after filtering calls with genotype quality less than 30, minor allele frequency less than 0.3, heterozygosity rate larger than 0.05, and variants for which less than 100 individuals were genotyped. Missing genotypes were imputed and parental assignment of individual haplotypes was obtained running the ImputeVCF command of NGSEP with the following parameters: ‘-p UCHaskell, UC92 -k 2 -c 0.003 -ip –is’. Recombination events between each pair of neighboring markers were identified and centimorgans were estimated assuming five generations of crossover and one expected crossover per individual chromosome per generation.

Ninety-three RILs and 10,497 polymorphic SNP markers were used to construct the genetic map using the ASMap v.1.0.4 and R/qtl v.1.44 packages in R^105,106^. Markers with less than 20% missing genotypes were used for map construction, and individuals with excessive recombination rates and more than 50% missing genotypes were removed from map construction. Linkage groups were formed using the ‘mstmap’ function with *p* < *e*-8 and genetic distances were calculated using the ‘Kosambi’ mapping function. Linkage groups were merged when originating from the same chromosome and recombination frequencies were recalculated by chromosome with lower *p*-values. Recombination rates across the genetic map were calculated using the ‘MareyMap’ function using the ‘sliding window’ interpolation method every 200 kbp in a 1 Mbp sliding window^107^. Pericentromeric regions of the chromosomes were defined when recombination rates exceeded 2 Mbp/cM for a given locus. Chromosome numbering followed the numbering of *P. vulgaris* chromosomes to obtain a one-to-one correspondence, justified by the high level of synteny^108^.

QTL mapping for plant habit, seed weight, days to first flower, and cyanogenesis was performed in the UC 92–UC Haskell RIL population using the R/qtl package^105^. A genome-wide scan for single QTLs was performed using the ‘scanone’ function with the extended Haley-Knott regression method^105^. For traits that were controlled by a single QTL, composite interval mapping was performed using the ‘cim’ function with the extended Haley-Knott regression method, the Kosambi mapping function, and 1000 permutations to identify the position and LOD score of QTLs above the 95% significance threshold. For traits that contained multiple significant QTL, the ‘makeqtl’ and ‘fitqtl’ functions were used to identify the optimal multiple QTL model, and to calculate LOD scores, percent of phenotypic variation explained by the QTL and QTL effects.

Days to first flower were collected from an experiment grown at two locations in Davis, California in 2018. The date was recorded when a majority of plants in the plot had at least one floral bud open. An ANOVA of a generalized linear mixed model, including genotype and treatment as fixed effect factors and location and blocks as random effect factors, was performed for days to first flower. Phenotypic characterization for plant habit, hundred seed weight, and delayed senescence included in the QTL mapping study were performed on single plants that were grown within a greenhouse.

Cyanide quantification of samples of floral bud tissue was collected in triplicate subsamples from an experiment grown at two locations in Davis, California in 2018. Tissue samples were kept on dry ice, weighed, and organized in 96-well plates and frozen at −80 °C. Cyanide quantification was performed using an adapted Fiegl-Anger method^109^. Tissue samples thawed at room temperature for 30 min before Fiegl-Anger paper was placed over each plate for 30 min. Standards of hydrogen cyanide were created with concentrations of 0, 25, 50, 75, 100, 250, 500, 750, and 1000 nM of potassium cyanide exposed to Fiegl-Anger paper for 30 min. Fiegl-Anger paper was immediately scanned, and quantification of the blue absorbance intensity was calculated using ImageJ software and the ‘readplate2’ plugin. Blue absorbance intensity was calculated as -log(Mean/255). A standard regression curve was calculated from the standard HCN concentrations and nanomolar concentrations for the samples were calculated based on this curve and calculated as an nM/min rate across the initial 30-min interval measured. Two separate linear models and ANOVAs were performed for the RIL population grouped by haplotype at the QTL peak on chromosome Pl05, since this QTL effect consistently produced bimodal distributions. The best fitting linear model for floral bud cyanogenesis included genotype, location, and treatment as fixed effect factors.

### Genetic diversity and population structure analyses

For GBS library construction, five biological samples of leaf tissue (three young trifoliate leaves of five different plants) were collected for each accession. The samples were frozen and stored at −20 °C until processing. DNA isolation was performed from frozen trifoliate leaves using the extraction method developed by Vega-Vela and Chacon Sanchez (2011)^110^. DNA quality was checked with a Nanodrop 2000 and analyzed in 1% agarose gel electrophoresis; DNA with no visible degradation and a ratio 260/280 above 1.8 was selected. DNA libraries (one per accession) were generated by mixing the DNA of five individuals in equal parts (with the help of a Nanodrop 2000 and electrophoresis calibration with *lambda* DNA at concentrations of 25, 50, and 100 ng/µL), then the DNA restriction for each sample was performed with the *Ape*KI enzyme and ligated to adapters containing one of 94 unique barcodes for each plate. The library was sequenced in paired end using an Illumina Hi-Seq2000. Both library preparation and sequencing were done at the Australian Genome Research Facility (Melbourne, Australia).

A total of 482 Lima bean accessions from the International Center for Tropical Agriculture—CIAT (262 accessions) and Centro de Investigacion Cientifica de Yucatan—CICY (220 accessions) were analyzed in this study (see Supplementary Data 3). Of these accessions, 215 were domesticated and 267 wild. Wild accessions covered the known natural geographic range for this species and domesticated accessions were landraces from different countries in the Americas (Supplementary Fig. 10). These accessions complemented those analyzed in a previous study^10^ to increase the sampling of wild accessions from the MI gene pool (the putative ancestral gene pool of Mesoamerican landraces) and gene pool AII from central Colombia. Sequenced accessions from CIAT are available through the mechanisms established by the germplasm collection. Sequenced accessions from Centro de Investigacion Cientifica de Yucatan (CICY) are available directly upon request.

The sequences obtained from each sample were de-multiplexed by their barcode using Next Generation Sequencing Experience Platform (NGSEP) v.3.3.2^85^. Individual fastq files were mapped to the Lima bean reference genome by using default parameters of bowtie2 and adding an ID code for each accession^84^. Variants were identified and individuals were genotyped using the MultisampleVariantsDetector command of NGSEP. The parameters used for SNP calling were: 50 as a maximum number of alignments per start position, heterozygosity rate of 0.0001 (prior probability of finding in every position a heterozygous SNP), and a minimum genotype quality score of 40 (codified in Phred, where 40 means 0.9999 of posterior probability that each genotype call is correct). Initial filtering to obtain a raw set of reliable variants was obtained by filtering using the FilterVCF command of NGSEP with the following criteria: (1) Exclude variants from repetitive regions and in not-assembled scaffolds; (2) Exclude variants genotyped in less than 100 of the 482 accessions; and (3) Exclude variants with minor allele frequency (MAF) less than 0.01. This procedure generated an initial set of 116,030 biallelic SNVs, 7517 biallelic indels and STRs, and 22,138 multiallelic variants (96.6% of them triallelic SNVs) with approximately 40% of missing data. Further filtering was applied for the different downstream population genetics analyses (details below).

Linkage disequilibrium (LD) was measured as r^2^, the correlation coefficient among pair of alleles across pairs of SNP loci, specifically as average r^2^ against 10 and 100 kbp physical intervals through the 11 chromosomes of *Phaseolus lunatus* using TASSEL v.5^111^. This analysis was conducted on a filtered dataset with the wild samples, as well as a filtered dataset with the domesticated samples. We also conducted the analysis for Mesoamerican and Andean gene pools. Results were plotted in R software by using an adapted code.

To estimate the number of clusters in Lima bean useful to describe the genetic data, we used algorithmic approaches and model-based approaches. For this, we built a multilocus-genotype matrix containing the 482 accessions (267 wild and 215 domesticated) described earlier. The raw VCF file described in the previous section was filtered keeping only biallelic SNVs with a distance of more than 10 bp from other variants, genotyped with quality at least 40 in at least 450 individuals and with minor allele frequency larger than 0.03. A total of 12,398 SNVs were retained after these filters with only 5.3% of missing data. For each sample, genotype calls based on read data were present for at least 75% of the SNPs and this percentage is less than 90% for only six samples (Supplementary Data 4).

The algorithmic approaches were carried out with the software Darwin v.6.0.021^112^ and the R package Adegenet v.2.1.2^113^. In Darwin, a pairwise distance matrix was calculated by estimating the proportion of matching alleles per locus for every pair of individuals. For the representation of the genetic structure, we used three methods: a principal coordinate analysis (PCoA in Darwin), a tree method (neighbor-joining -NJ- algorithm in Darwin), and a discriminant analysis of principal components^114^ (DAPC in Adegenet), that unlike PCoA, optimizes the variance between groups while minimizing the variance within groups. In Adegenet, the function ‘find.clusters’ was used to identify the optimal number of clusters based on the Bayesian information criterion (BIC), 500 principal components were retained, four discriminant functions were used and the proportion of conserved variance was 0.95. For statistical support of NJ tree structure, 1000 bootstrapped matrices were obtained. The tree was visualized using iTol^103^.

The software STRUCTURE v.2.3.4^115^ was used as an alternative approach to infer the population structure of the sequenced accessions. The filtered VCF file was converted to the input format of STRUCTURE using the ConvertVCF command of NGSEP. A total of 200 STRUCTURE runs were executed varying the number of clusters (*K* parameter) from 1 to 10 and performing 20 repetitions for each *K* value. For each run, 10,000 burn-in iterations and 30,000 sampling iterations were executed. The main and extra parameters were left with default values with the exception of the option ‘ONEROWPERIND’ which was set to 1 to make it consistent with the format provided by the VCF converter of NGSEP. Stability of the Markov chain was assessed looking at the variance of the likelihood of the clustering achieved by each repetition. The solution with the best likelihood was selected for each *K* value between 2 and 8 and cluster numbers were reorganized to visually assess the cluster stability across different *K* values (Fig. 3a). The Evanno test was performed to predict the optimal number of clusters^116^.

Haplotype based population structure was assessed with the software fineSTRUCTURE v.4.1.0^57^. In brief, this software builds a sample relationship matrix capturing the haplotype similarity across the genome. This matrix is then used to run a Markov chain to sample group configurations and tree topologies consistent with the similarity matrix. For this analysis, the VCF file with population information was filtered keeping only biallelic SNVs with a distance of more than 10 bp from other variants, genotyped with quality at least 40 in at least 300 individuals, MAF larger than 0.02, and observed heterozygosity (OH) below 0.5. A total of 28,753 SNVs were retained after these filters with 11% missing data and 332,817 (2.4%) heterozygous data points. Imputation of missing data and statistical haplotyping was performed using Beagle 5.0^117^ setting the phase states to 50, the imputation states to 400, and the effective population size to 100,000. For each chromosome, the VCF was converted to the input format of fineSTRUCTURE using the command VCFConverter of NGSEP. The four steps pipeline of fineSTRUCTURE was executed using default parameters following the user manual and providing genetic distances inferred from the genetic map developed from the biparental population.

Basic diversity statistics for populations, namely observed heterozygosity (*H*_O_), expected heterozygosity (*H*_E_), endogamy (*F*_IS_), global *F*_ST_, and pairwise *F*_ST_^118^ were estimated using the R packages Adegenet^113^ and Hierfstat v.0.5^119^ from the same dataset used to run STRUCTURE. Difference between *H*_O_ and *H*_E_ was assessed by means of the Bartlett test and paired t test. The significance of population differentiation was tested by means of a likelihood ratio G-statistic^120^.

Introgression analysis was performed running the ‘IntrogressionAnalysis’ command of NGSEP. The input VCF for this analysis was the same used to run STRUCTURE. The input file with population assignments contained genetic populations inferred by STRUCTURE at *K* = 4, which separates the MI, MII, AI, and AII populations. Only 448 samples with clear assignments were included in this file. The analysis was executed with default parameters (non-overlapping windows of 50 SNPs discriminating at least one pair of populations). The raw set of predicted introgression events called with this procedure was filtered keeping only events that span more than one contiguous window or, for a single window of 50 SNPs, if the similarity score between the haplotype of the sample and the consensus haplotype of the sample background population was below 15. Then, the D-statistic implemented in the ANGSD package v.0.93^121^ was calculated to assess significance of the introgression events. GBS reads from common bean were aligned to the *P. lunatus* genome to use them as an outgroup for this analysis. Events with Z-scores larger than 2 were retained. Additionally, the introgression called for the MII sample JMC_1097 was retained because it spanned 53Mbp over four chromosomes. Finally, two introgressions of about 2Mbp called at 36 Mbp of Pl07 and 4.6Mbp of Pl08 were retained because they were consistently called in six and seven accessions, respectively. Although the D-statistic probably did not have enough power to assess significance of these introgressions, they were validated by construction of neighbor-joining clusters within the specific regions (Supplementary Fig. 22).

### Reporting Summary

Further information on research design is available in the Nature Research Reporting Summary linked to this article.

## Supplementary information

Supplementary Information

Peer Review File

Reporting Summary

Description of Additional Supplementary Files

Supplementary Data 1

Supplementary Data 2

Supplementary Data 3

Supplementary Data 4

Supplementary Data 5

Supplementary Data 6

Supplementary Data 7

## Data Availability

Data supporting the findings of this work are available within the paper and its Supplementary Information files. A reporting summary for this article is available as a Supplementary Information file. The datasets and plant materials generated and analyzed during the current study are available from the corresponding author upon request. Accession codes and passport data for the 482 wild and domesticated accessions analyzed in this study are shown in Supplementary Data 3 (CIAT’s and CICY’s accession numbers). Accessions from CIAT can be requested at https://genebank.ciat.cgiar.org. Raw sequence data generated in this study have been deposited in the NCBI Sequence Read Archive (SRA) under the Bioproject accession number PRJNA596114. The genome assembled in this study is available at the NCBI assembly database with accession number JAAFYQ000000000. The genome assembly and its corresponding annotation are also available at Phytozome v.13. Other relevant data are available in Supplementary Data files. We reanalyzed RNA-seq data publicly available at NCBI SRA with bioproject accession number PRJNA275266. Source data are provided with this paper.

## Source data

Source Data

## Code availability

Software developed for this work is available as part of the open-source software solution NGSEP. Stable releases of NGSEP are available through sourceforge [http://ngsep.sf.net]. Live development is constantly made available through our github repositories [http://github.com/NGSEP].
