# Supplementary material for: Comprehensive genomic resources related to domestication and crop improvement traits in Lima bean
Source: Nat Commun. 2021 Jan 29;12:702. doi: 10.1038/s41467-021-20921-1 (PMC7846787; doi:10.1038/s41467-021-20921-1)
Supplement: Supplementary file 1 — Supplementary Information [file 41467_2021_20921_MOESM1_ESM.pdf]

Comprehensive genomic resources related to domestication and breeding traits in Lima bean

Garcia *et al*.

Supplementary Table 1. Summary of genetic markers, genetic and physical distances mapped to each Lima bean chromosome.

Chr	No Markers	Loci	First Marker (bp)	Last Marker (bp)	Physical Coverage (Mbp)	Genetic Length (cM)	Max Dist Between Loci (cM)	Avg Dist Between Loci (cM)	Avg Dist Between Loci (kbp)
Pl01	119	27	591,375	47,588,051	47	113.3	20.5	4.2	1740.62
Pl02	1554	60	720,985	51,607,776	50.89	136.5	24.4	2.27	848.11
Pl03	1102	68	2,903	45,042,368	45.04	116.9	7.8	1.72	662.35
Pl04	887	45	66,315	49,299,606	49.23	91.4	12.2	2.03	1094.07
Pl05	1263	43	24,078	38,089,793	38.07	74.7	7.5	1.74	885.25
Pl06	661	46	2,763	36,649,143	36.65	60.3	3.5	1.31	796.66
Pl07	1604	56	1,778	47,820,557	47.82	98.4	8.2	1.76	853.91
Pl08	749	73	35,793	57,107,478	57.07	109.8	6.7	1.5	781.8
Pl09	310	43	2,050	40,853,806	40.85	133.7	32.8	3.11	950.04
Pl10	453	26	53,329	53,972,946	53.92	62	11.4	2.38	2073.83
Pl11	1795	35	30,471	48,067,523	48.04	66.7	9	1.91	1372.49
Total	10497	522			514.57	1063.8	144		
Mean	954.3	47.5	139,258.18	46,918,095.18	46.78	96.7	13.1	2.18	1096.28

Source data are provided as a Source Data file.

Supplementary Table 2. Recombination rates across euchromatic and pericentromeric regions for each chromosome.

Chr	Chromosome (cM/Mbp)	Euchromatic Arms (cM/Mbp)	Euchromatic Short Arm (cM/Mbp)	Euchromatic Long Arm (cM/Mbp)	Pericentromere (cM/Mbp)	Start of Pericentromere (Mbp)	End of Pericentromere (Mbp)	Pericentromere Length (Mbp)
Pl01	2.41	8.62	7.52	9.81	0.1	7.72	22.11*	14.38*
Pl02	2.68	5.43	5.29	5.51	0.16	9.984	36.417	26.43
Pl03	2.6	3.83	5.23	3.45	0.15	6.03	23.711	17.68
Pl04	1.86	7.08	5.43	9.66	0.09	7.927	44.546	36.62
Pl05	1.96	4.63	4	5.35	0.15	8.325	30.802	22.48
Pl06	1.65	3.42	--	3.42	0.2	0.003	19.761	19.76
Pl07	2.06	4.78	5.5	4.21	0.27	8.422	37.151	28.73
Pl08	1.92	4.29	4.48	4.11	0.23	14.141	45.172	31.03
Pl09	3.27	6.24	6.61	5.98	0.56	8.18	29.51*	21.33*
Pl10	1.15	5.43	4.15	6.93	0.1	5.766	49.1	43.33
Pl11	1.39	6.04	8.38	3.6	0.09	5.414	42.758	37.34
Total								299.12
Mean	2.1	5.4	5.7	5.6	0.2			27.19
*denotes region of limited marker coverage and potentially inaccurate estimate.								

Source data are provided as a Source Data file.

Supplementary Table 3. Summary of repetitive elements identified in the Lima bean genome assembly.

Class	Number of Elements	Length	Percentage of sequence
SINE	2,299	231,813	0.04%
LINE	24,691	7,861,362	1.44%
LTR	322,004	174,499,500	31.94%
DNA	72,189	25,254,401	4.62%
Unclassified	30,886	6,025,648	1.10%
Total interspersed repeats	452,069	213,872,760	39.14%
Simple repeats	163,489	9,028,075	1.65%
Low complexity	41,370	2,333,561	0.43%

Source data are provided as a Source Data file.

Supplementary Table 4. QTL peaks, LOD scores, % variation explained and QTL effect for select traits in the UC 92 x UC Haskell population.

Trait	QTL Peak	LOD	% variation explained	QTL effect
Determinacy	Pl01_42432553	78.6	78.7%	--
Hundred Seed Weight (g)	Pl10_39674709	8.32	12.5%	6.2
	Pl03_26637359	4.3	6.2%	3.7
	Pl09_6453659	3.66	5.2%	3.4
	Pl04_2884774	3.28	4.7%	3.4
Flowering time	Pl01_42432553	9.8	29.9%	5.3
Cyanogenesis	Pl05_35651614	68.3	93.3%	11.7
	Pl10_44773521	9.98	2.8%	3.2
	Pl08_2715645	5.98	1.5%	-1.2
Seed coat color	Pl07_46475134	11.6	20.3%	--
	Pl07_45067785	4.5	8.5%	--

Source data are provided as a Source Data file.

Supplementary Table 5. Basic diversity statistics of gene pools in wild and domesticated Lima bean calculated on the basis of 454 accessions and 12,398 SNP markers.

**Population**	**N**	**H_E**	**H_O**	**F_{IS}**	**Founder effect**
All samples	454	0.32	0.03	0.85	
Wild	250	0.23	0.05	0.76	
Domesticated	204	0.17	0.01	0.95	0.25
Wild MI	110	0.13	0.08	0.53	
Dom MI	154	0.06	0.01	0.68	0.55
DOM MI (without landraces from Yucatan Peninsula)	93	0.07	0.01	0.68	
DOM MI (only landraces from Yucatan Peninsula)	61	0.03	0.01	0.8	
Wild AI	8	0.04	0.02	0.59	
Dom AI	34	0.04	0.01	0.84	0
Wild AII	18	0.05	0.01	0.7	
Wild MII	107	0.13	0.03	0.62	
Dom AII	2	0.11	0.03	0.58	
Dom MII	14	0.14	0.01	0.94	
Wild AI (Guatemala)	7	0.05	0	0.93	

Note: A total of 28 accessions that resulted to be admixed in the Structure analysis were not included. N: number of accessions. HE: expected heterozygosity. HO: Observed heterozygosity. FIS: endogamy index measured as mean over all loci. Founder effect: reduction in genetic diversity due to domestication measured as the percent difference in HE between wild and domesticated accessions. Source data are provided as a Source Data file.

Supplementary Table 6. Pairwise F_{ST} distance matrix among gene pools in wild and domesticated Lima bean, as measured on the basis of 451 accessions and 19,384 SNP markers.

	Dom MI	Dom MII	Dom AI	Wild MII	Wild MI	Wild AI	Wild AII	Dom AII
Dom MII	0.76	0						
Dom AI	0.86	0.81	0					
Wild MII	0.7	0.03	0.73	0				
Wild MI	0.33	0.54	0.72	0.54	0			
Wild AI	0.85	0.72	0.21	0.69	0.67	0		
Wild AII	0.83	0.61	0.88	0.56	0.65	0.86	0	
Dom AII	0.82	0.45	0.87	0.49	0.61	0.84	0.15	0

Source data are provided as a Source Data file.

Supplementary Table 7. Summary of sequencing data used to generate the *de novo* genome assembly.

Technology	Protocol	Number of reads (Million)	Raw data (Gbp)
Pacbio	SMRT	2	25.6
Illumina	Paired-end	2 x 103	31
Illumina	Linked reads (10x)	2 x 138	41
Total		243	97.6

Source data are provided as a Source Data file.

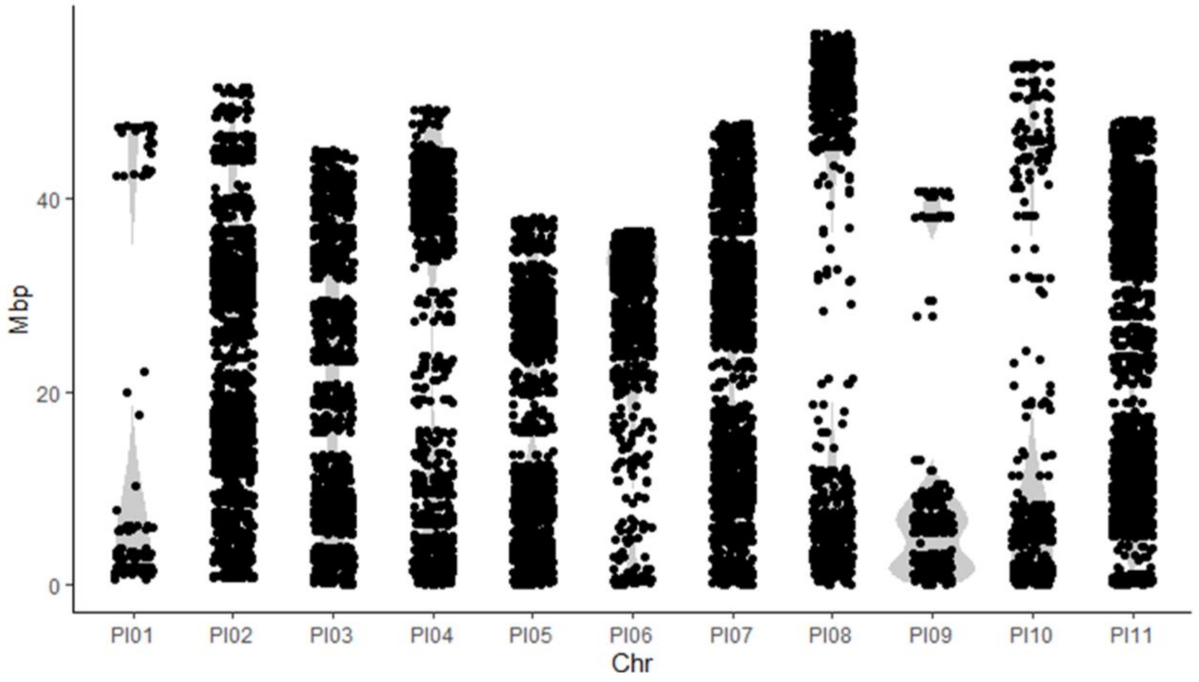

Supplementary Figure 1. SNP density for the biparental population. Visual representation of the physical locations of SNPs genotyped to build a genetic map from GBS data of the UC 92 x UC Haskell RIL population. Source data are provided as a Source Data file.

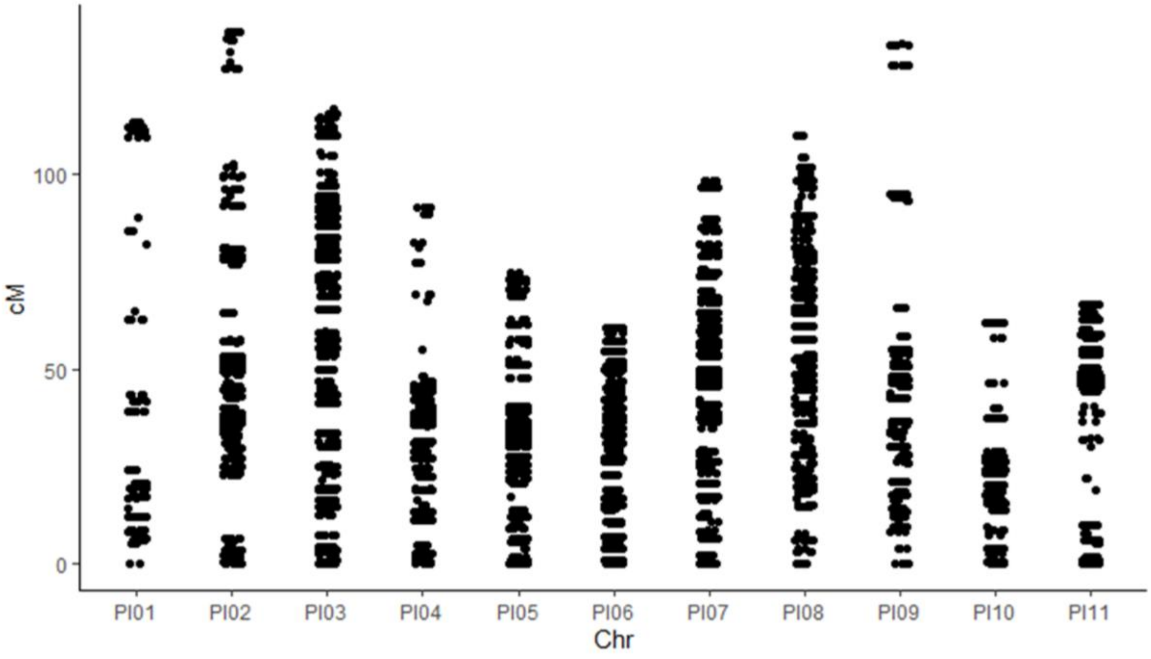

Supplementary Figure 2. Genetic distances for the biparental population. Locus distribution on the *Phaseolus lunatus* genetic map for the UC 92 x UC Haskell RIL population. Source data are provided as a Source Data file.

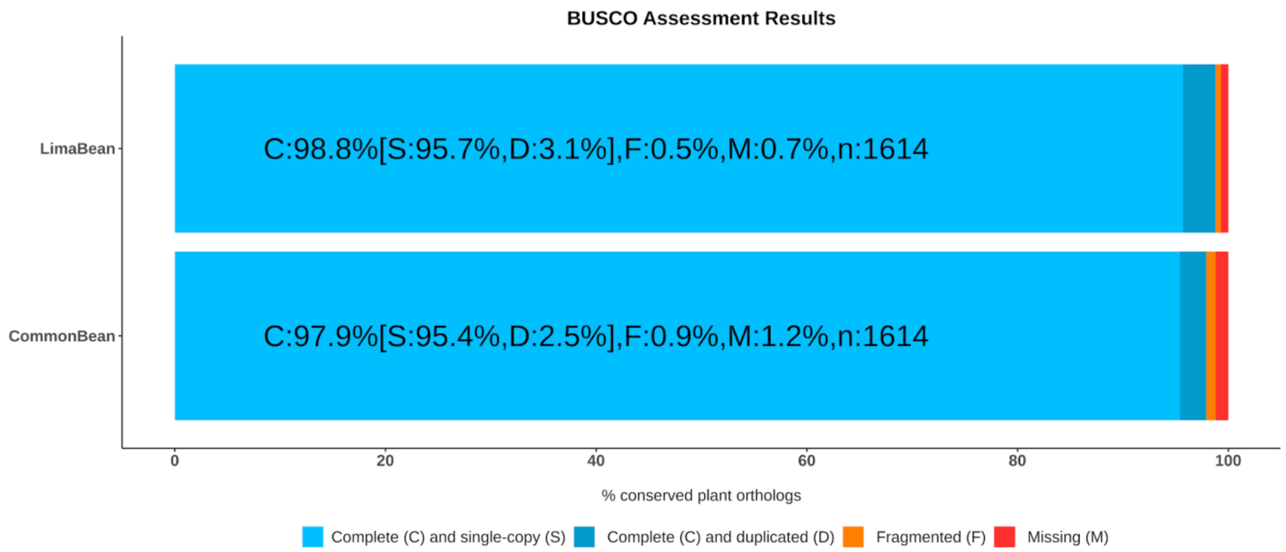

Supplementary Figure 3. Completeness of conserved gene models. Percentage of conserved genes in the plant kingdom (assessed by Busco) that can be found in the Lima Bean (*Phaseolus lunatus*) and common bean (*Phaseolus vulgaris*) genomes. Source data are provided as a Source Data file.

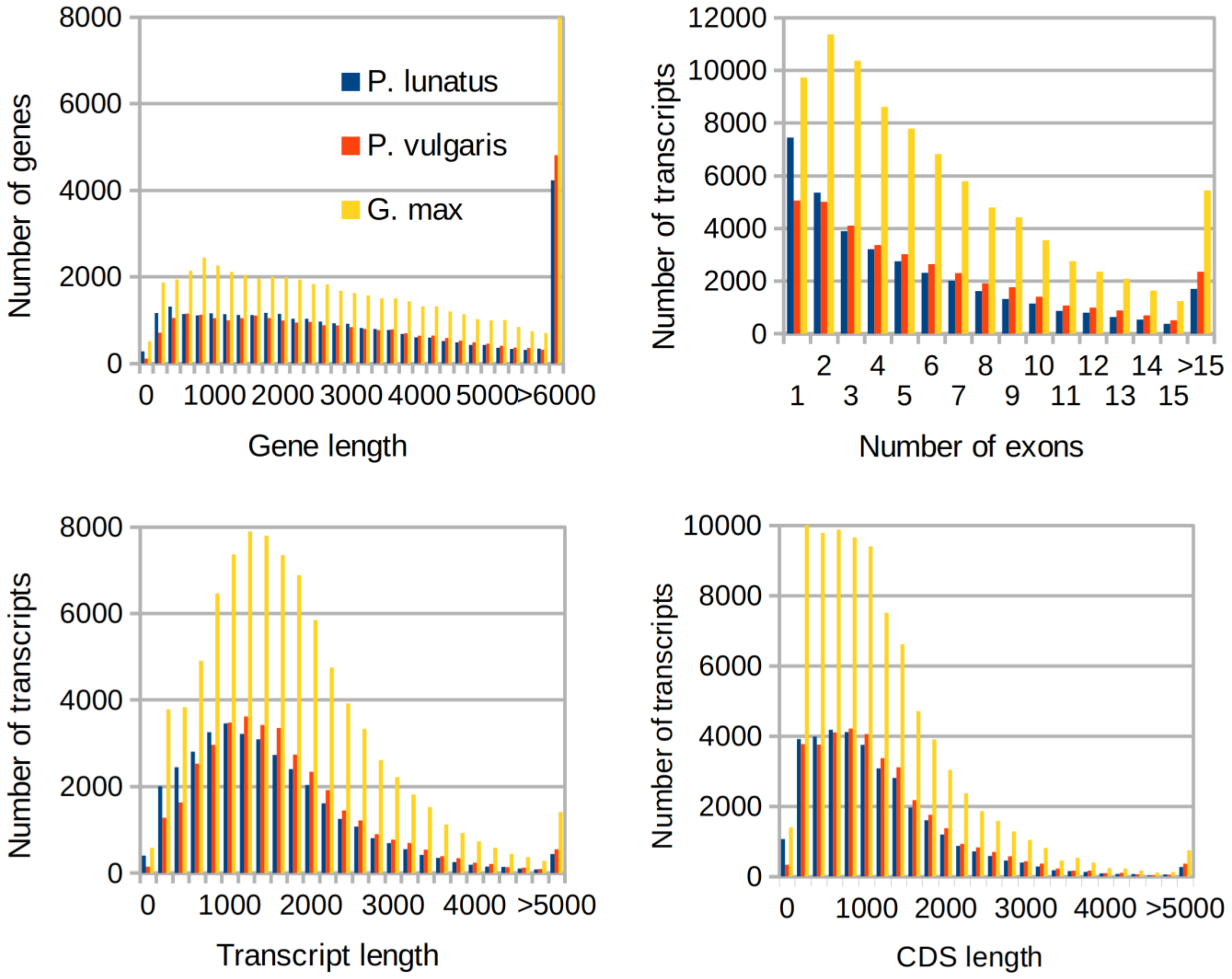

Supplementary Figure 4. Gene model annotation statistics. Distribution of gene, exon, transcript and CDS lengths, for annotated genes in the *P. lunatus* genome. The same distributions for *P. vulgaris* and *G. max* (Soy bean) are shown for comparison. Source data are provided as a Source Data file.

A.

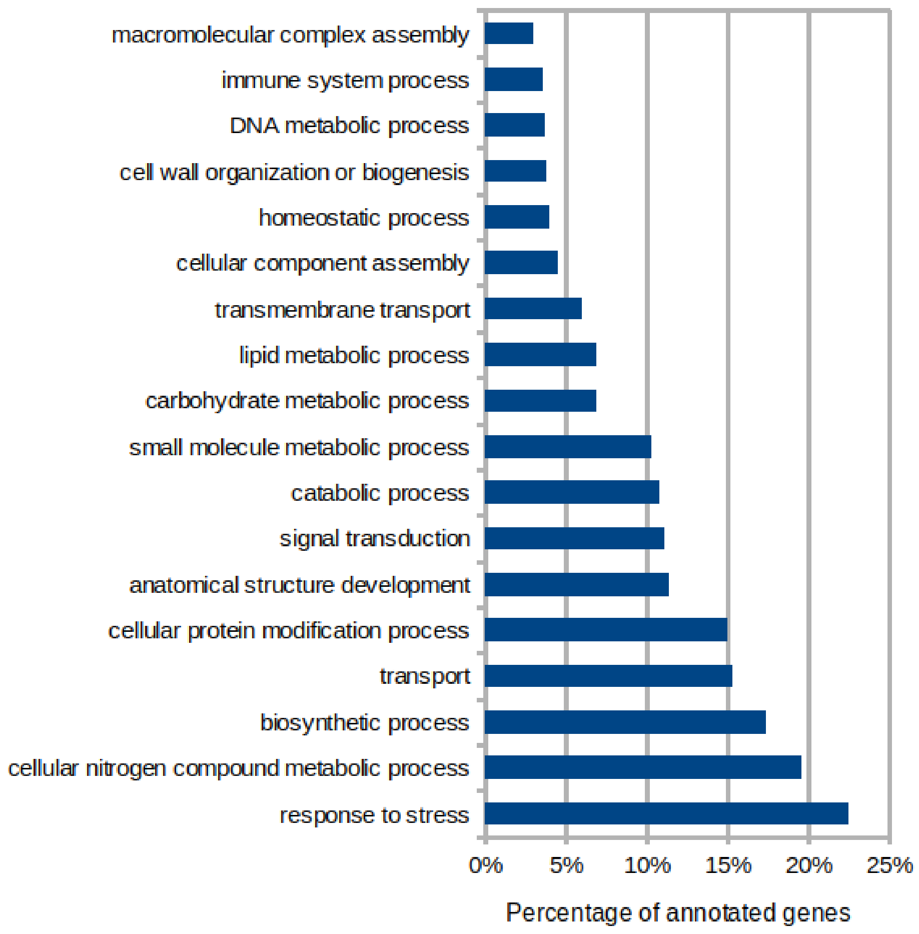

B.

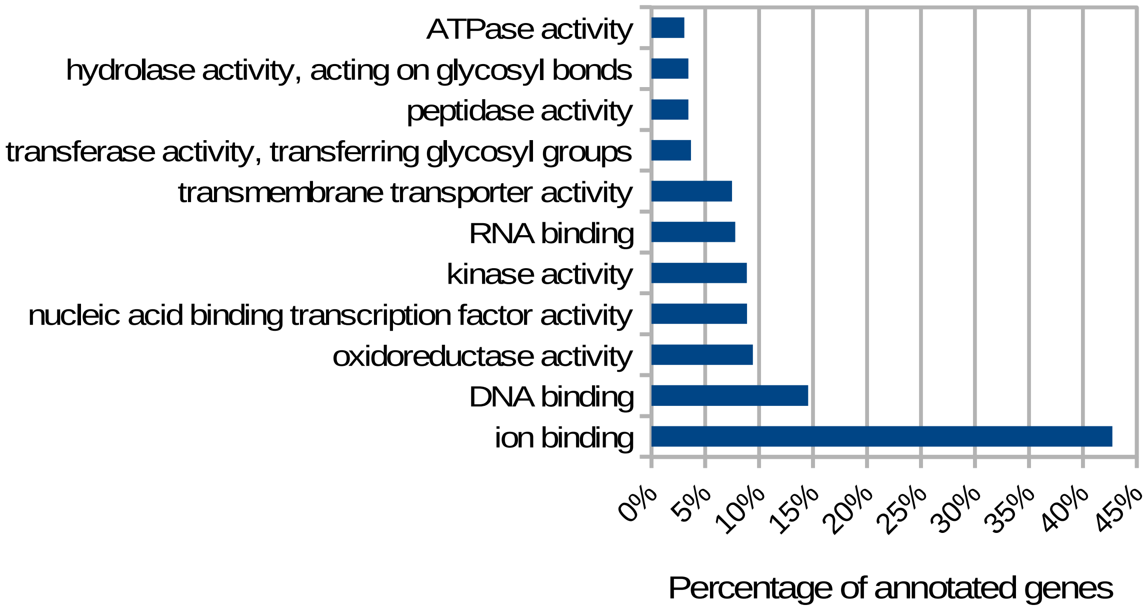

Supplementary Figure 5. Gene functional annotation. Gene ontology annotations assigned to at least 3% of the gene models. A. Biological processes. B. Molecular functions. Source data are provided as a Source Data file.

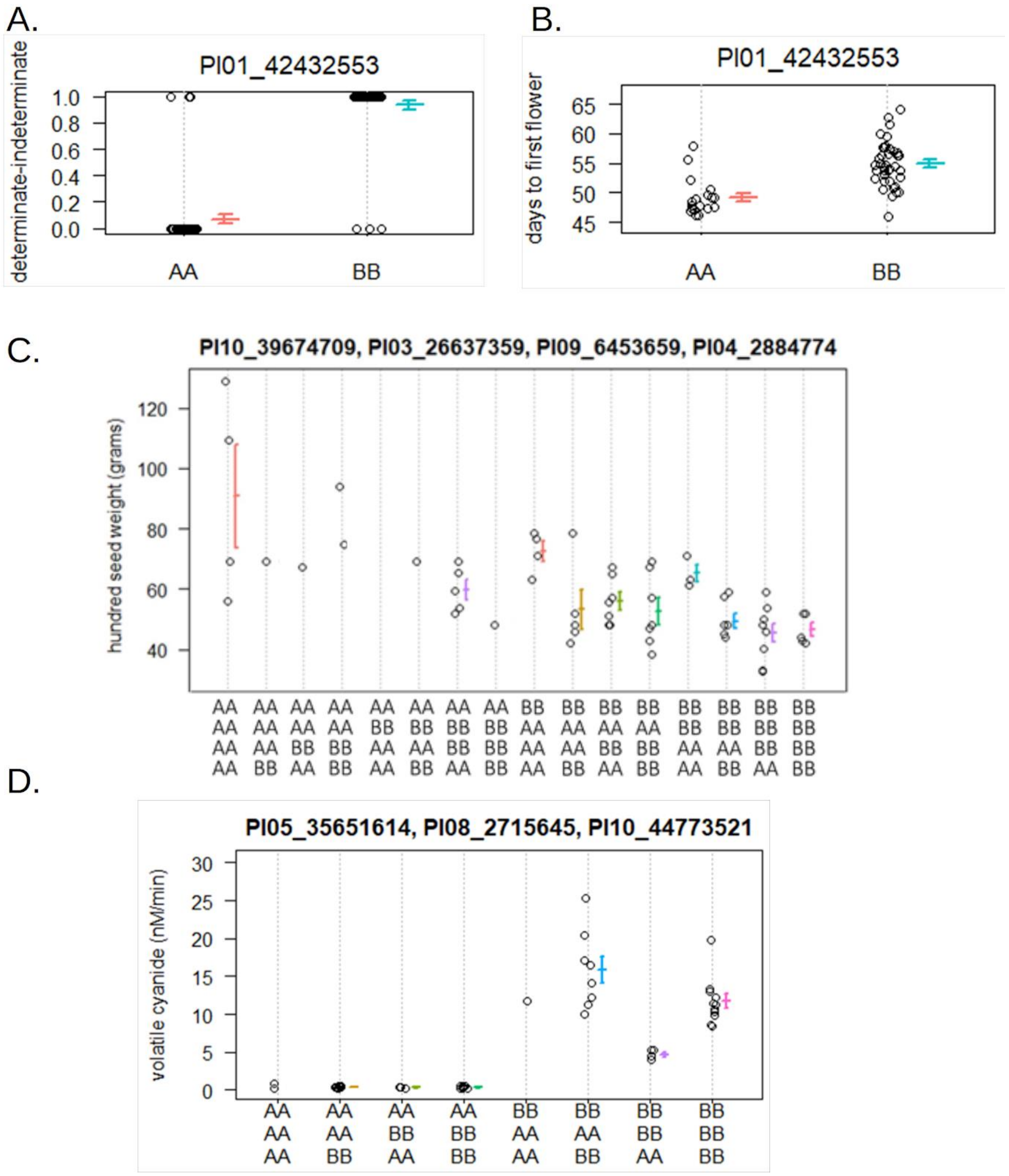

Supplementary Figure 6. Effects for significant QTL for different traits segregating in the UC Haskell – UC 92 population. Open circles represent individual progeny lines of this population. For each trait and each segregation class, colored brackets represent averages and lines represent standard error around the average. For each trait, 'AA' represents the UC 92 parental genotype and 'BB' represents the UC Haskell parental genotype. A. Determinacy scored as 0 for determinacy (inherited from parent UC 92) and 1 for indeterminacy (from parent UC Haskell. N (AA) = 43; N (BB) = 61. B. Days to first flower. N (AA) = 18; N (BB) = 38. C. Seed weight, measured as 100-seed weight. N(AA,AA,AA,AA) = 4; N (AA,AA,AA,BB) = 1; N(AA,AA,BB,AA) = 1; N(AA,AA,BB,BB) = 2; N(AA,BB,AA,AA) = 0, N(AA,BB,AA,BB) = 1; N(AA,BB,BB,AA) = 5; N(AA,BB,BB,BB) = 1; N(BB,AA,AA,AA) = 4; N(BB,AA,AA,BB) = 5; N(BB,AA,BB,AA) = 7; N(BB,AA,BB,BB) = 7; N(BB,BB,AA,AA) = 3;N(BB,BB,AA,BB) = 7; N(BB,BB,BB,AA) = 9; N(BB,BB,BB,BB) = 5. D. Volatile cyanide content of floral bud tissue. N(AA,AA,AA) = 2; N (AA,AA,BB) = 11; N(AA,BB,AA) = 3; N(AA,BB,BB) = 9; N(BB,AA,AA) = 1; N(BB,AA,BB) = 8; N(BB,BB,AA) = 4; N(BB,BB,BB) = 11. Source data are provided as a Source Data file.

A.

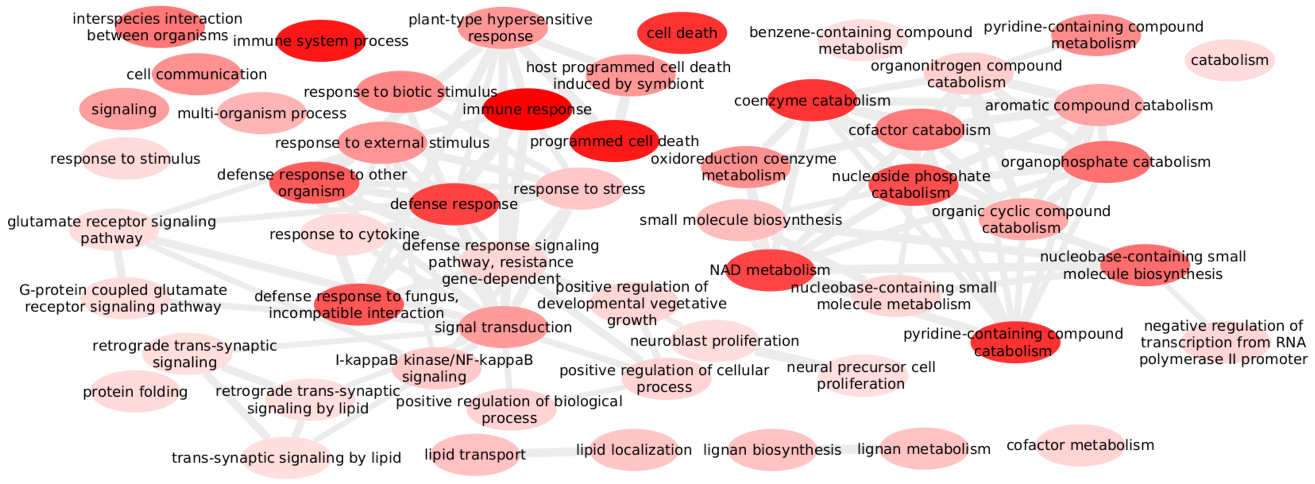

B.

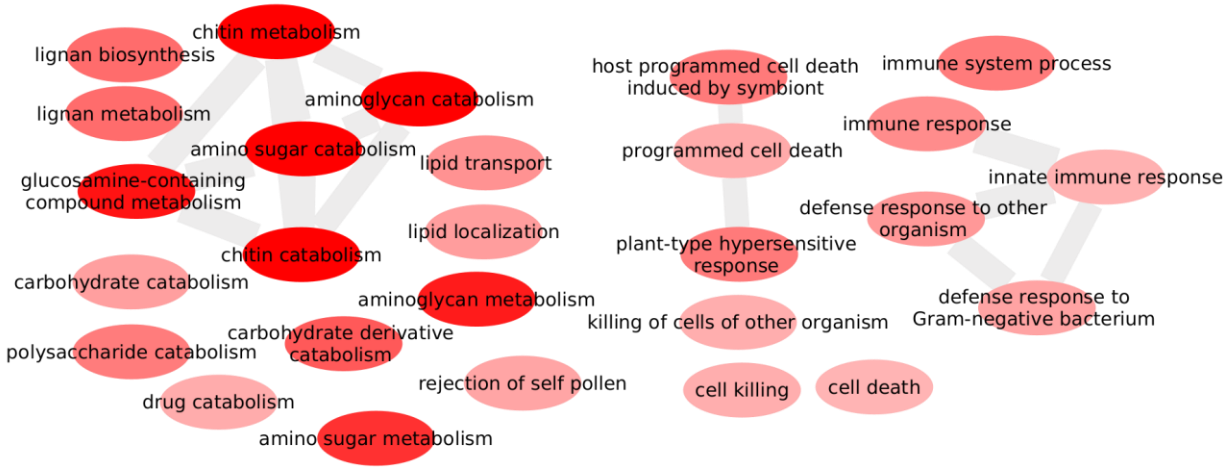

Supplementary Figure 7. Functional enrichment of homologs with high Ka/Ks values. Enrichment of GO terms in same chromosome paralogs (A) and synteny orthologs (B) with Ka/Ks values above 1. Source data are provided as a Source Data file.

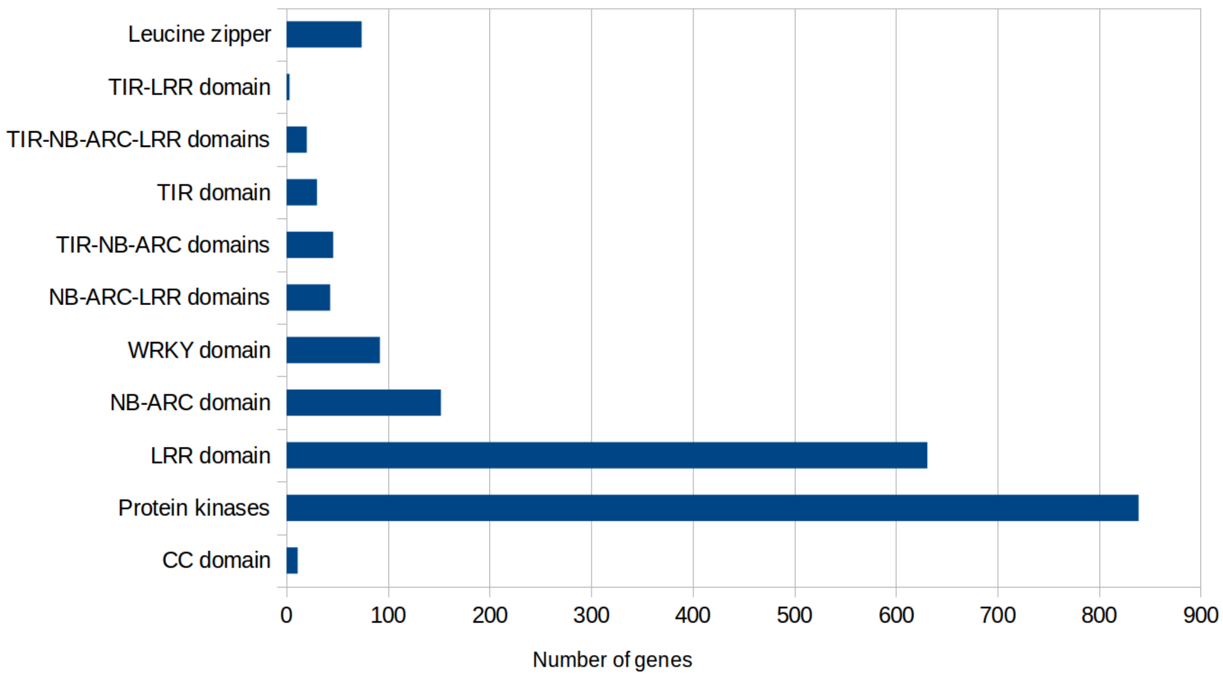

Supplementary Figure 8. LRR gene statistics. Distribution of number of genes with domains associated with resistance to biotic stress. Source data are provided as a Source Data file.

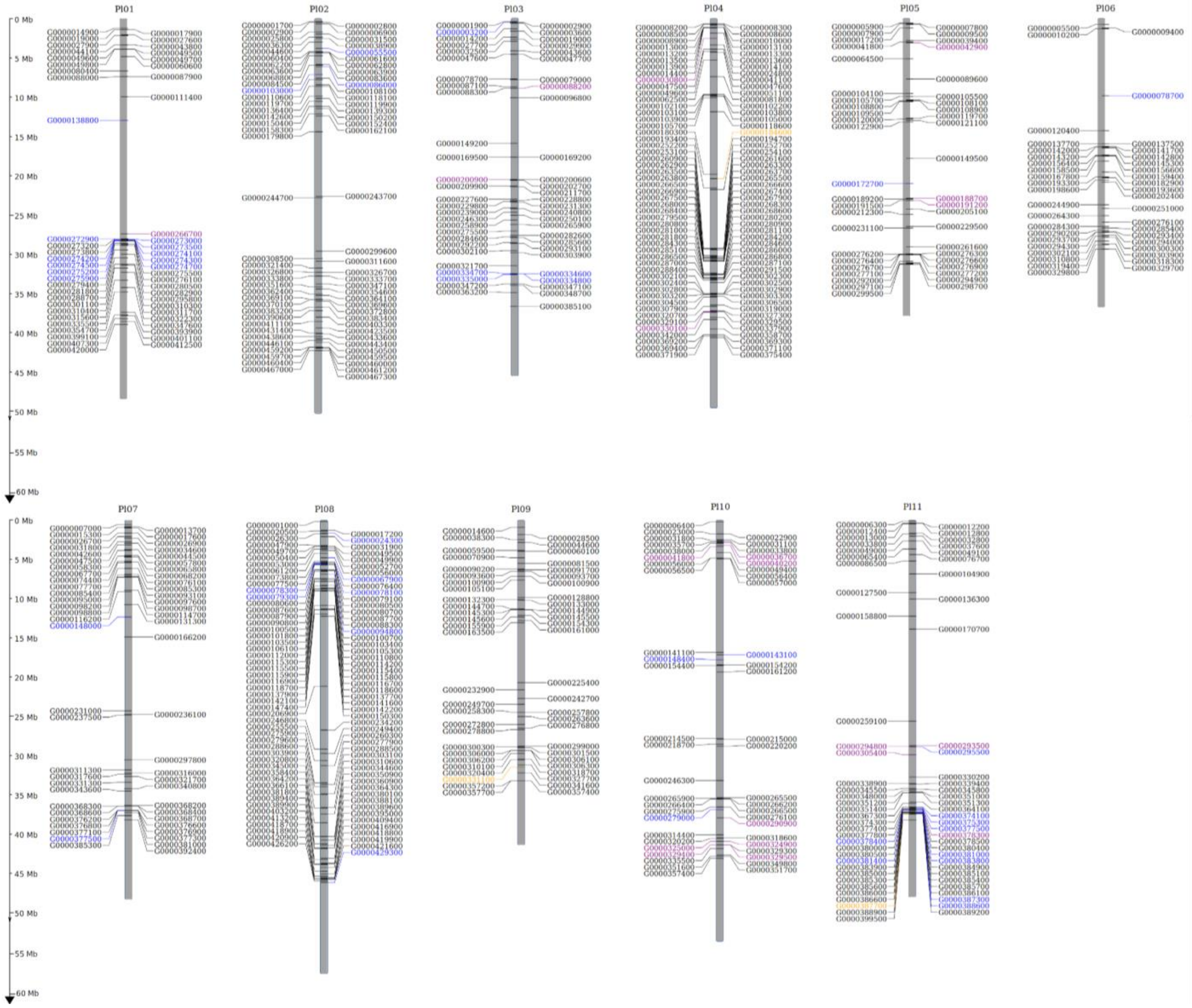

Supplementary Figure 9. Physical locations of genes with the LRR domain. Each gene has a unique label corresponding to the last 11 digits from the annotation ID (for example, gene G0000138800 on chromosome Pl01 corresponds to gene Pl01G0000138800). Black are genes only with the LRR domain, blue are genes with domains NB-ARC and LRR, purple are genes with domains TIR, NB-ARC and LRR, orange are genes with the TIR and LRR domains. Source data are provided as a Source Data file.

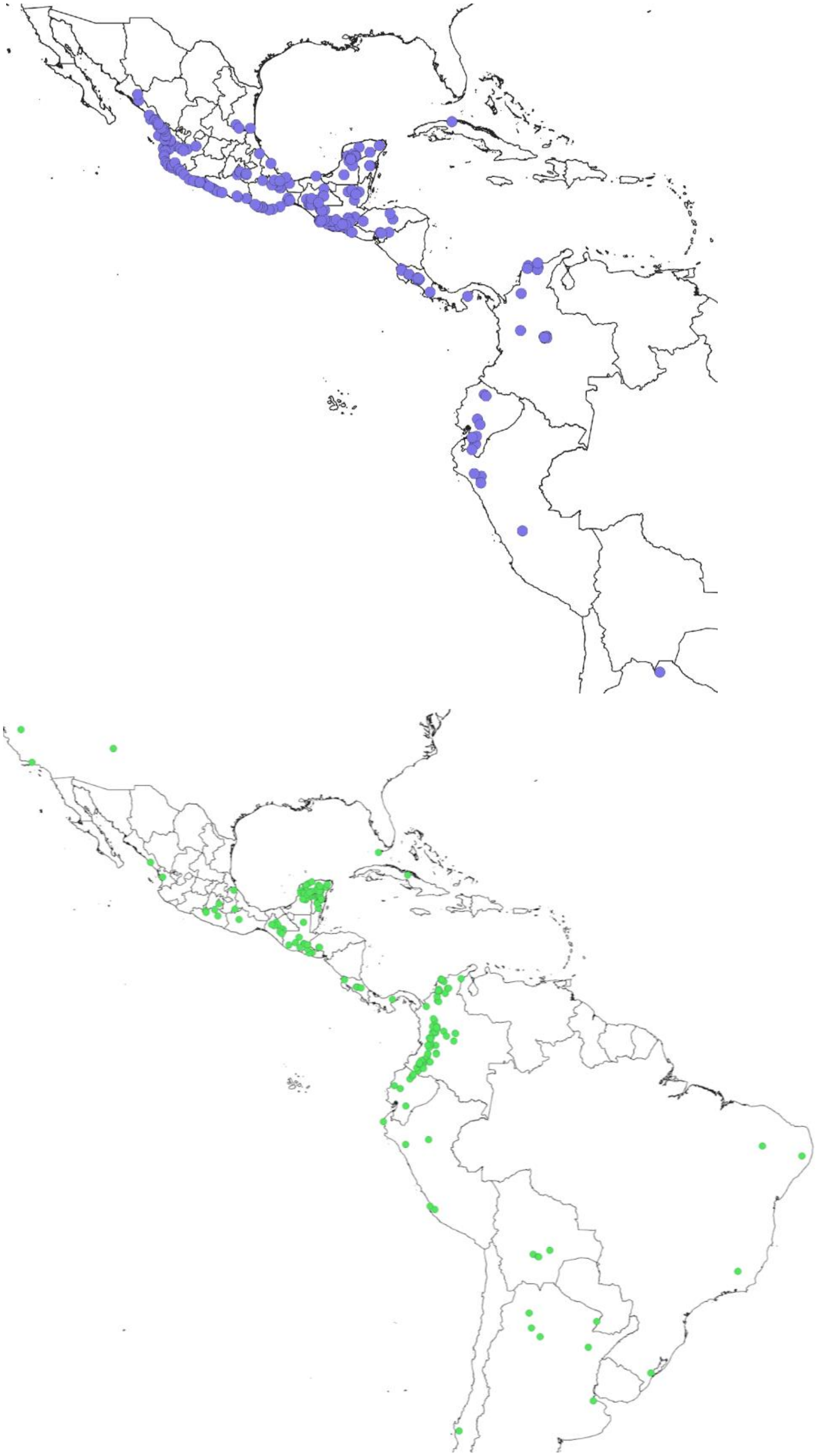

Supplementary Figure 10. General geographic distribution of accessions genotyped in this study. Wild accessions are shown in the top map and domesticated accessions are shown in the bottom map. Source data are provided as a Source Data file.

A.

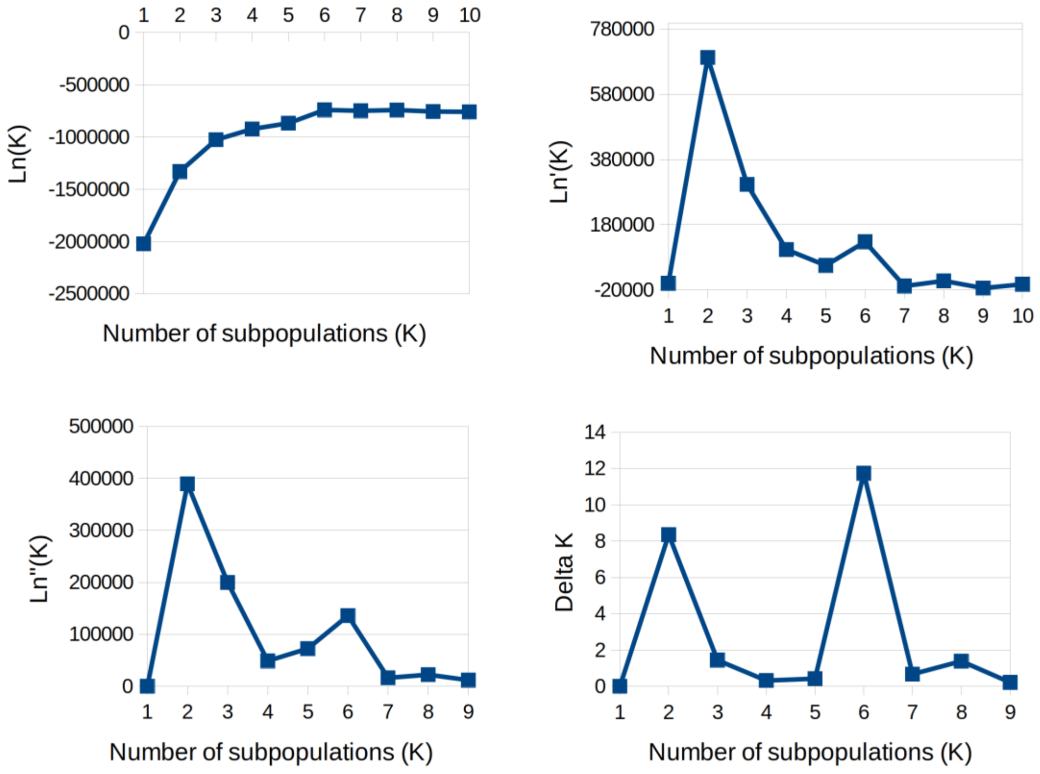

B.

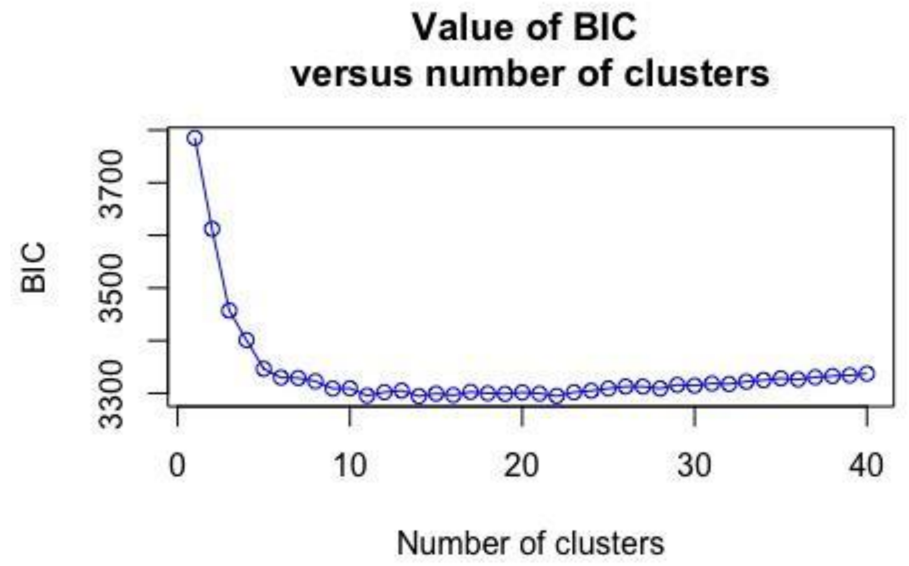

Supplementary Ffigure 11. Optimal number of cluster for structure analysis. Results of A. the Evanno test and B. The DAPC Bayesian information criterion test, to infer the optimal number of subpopulations. Source data are provided as a Source Data file.

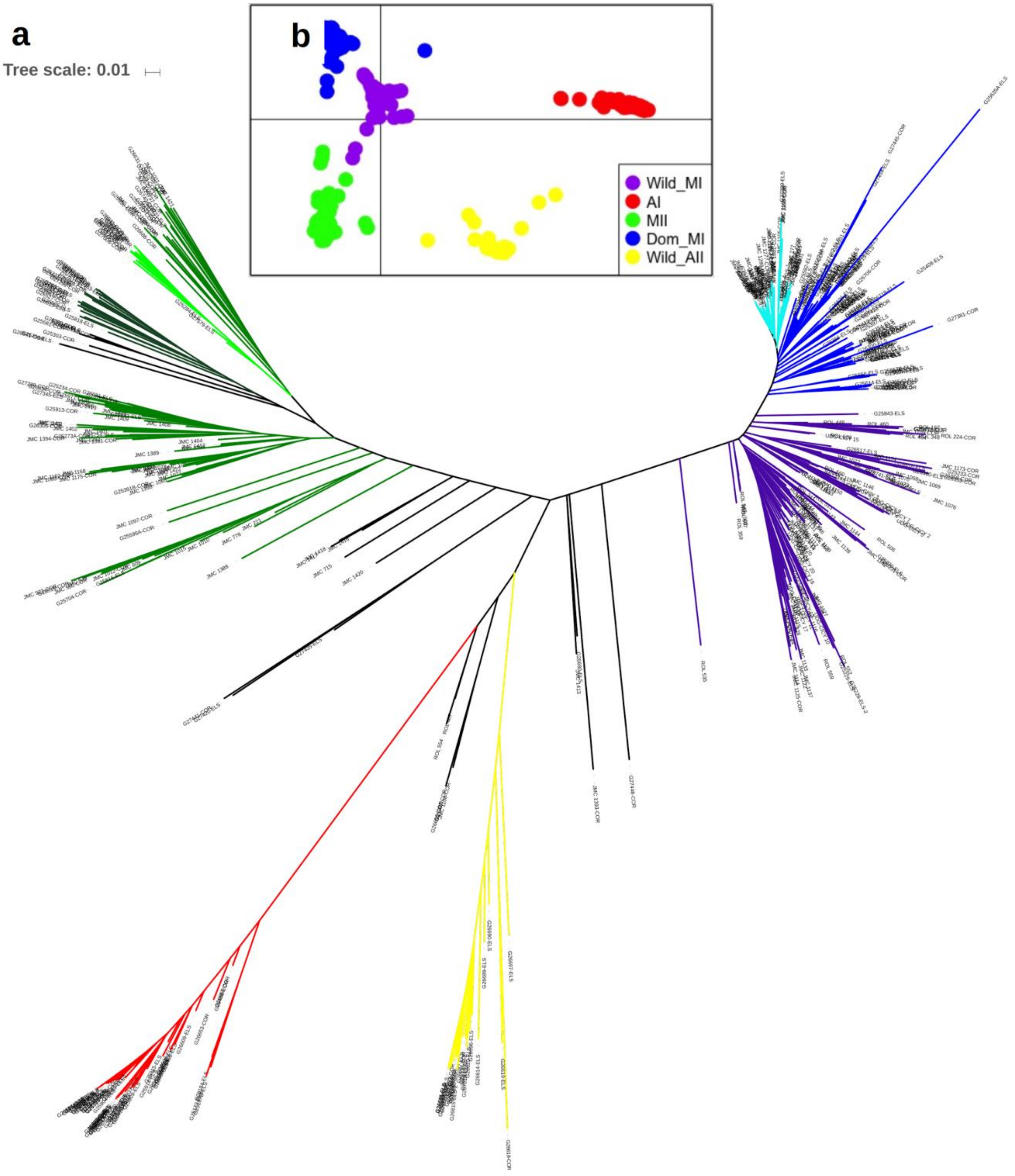

Supplementary Figure 12. Lima bean genetic variability. a. Neighbor-joining tree and **b.** discriminant analysis of principal components (DAPC) analysis of the genetic variability between 482 wild and domesticated Lima bean accessions collected across the Americas. Color coding for branches in the NJ tree is according to gene pools of accessions as followed: purple: wild MI, red: AI, dark green: wild MII, light green: domesticated MII, dark blue: domesticated MI, light blue: domesticated MI from Yucatan Peninsula and yellow: AII. Source data are provided as a Source Data file.

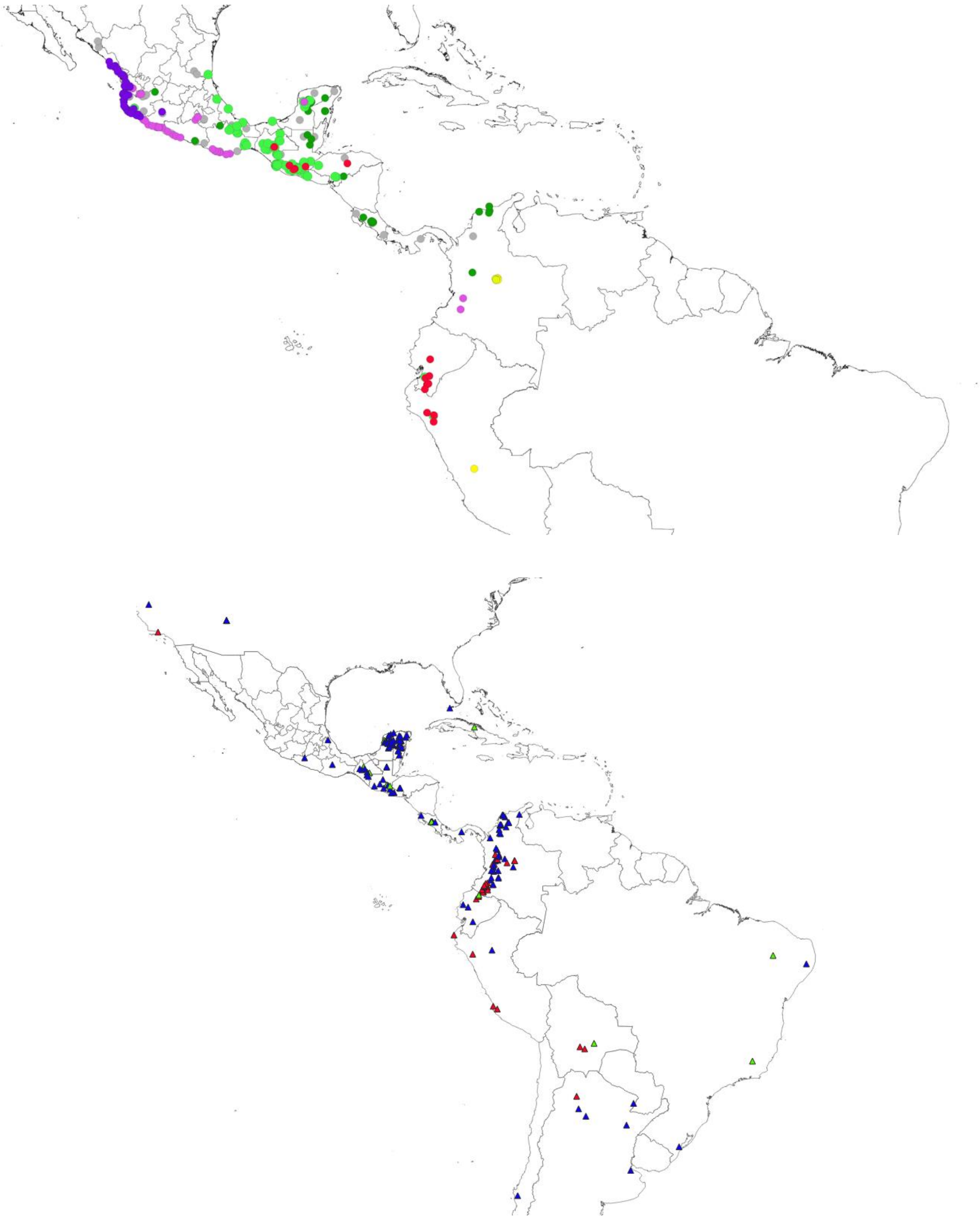

Supplementary Figure 13. Geographic distribution of accessions genotyped in this study, clustered according to the high level population structure (K=6). Wild accessions are shown in the top map and domesticated accessions are shown in the bottom map. Source data are provided as a Source Data file.

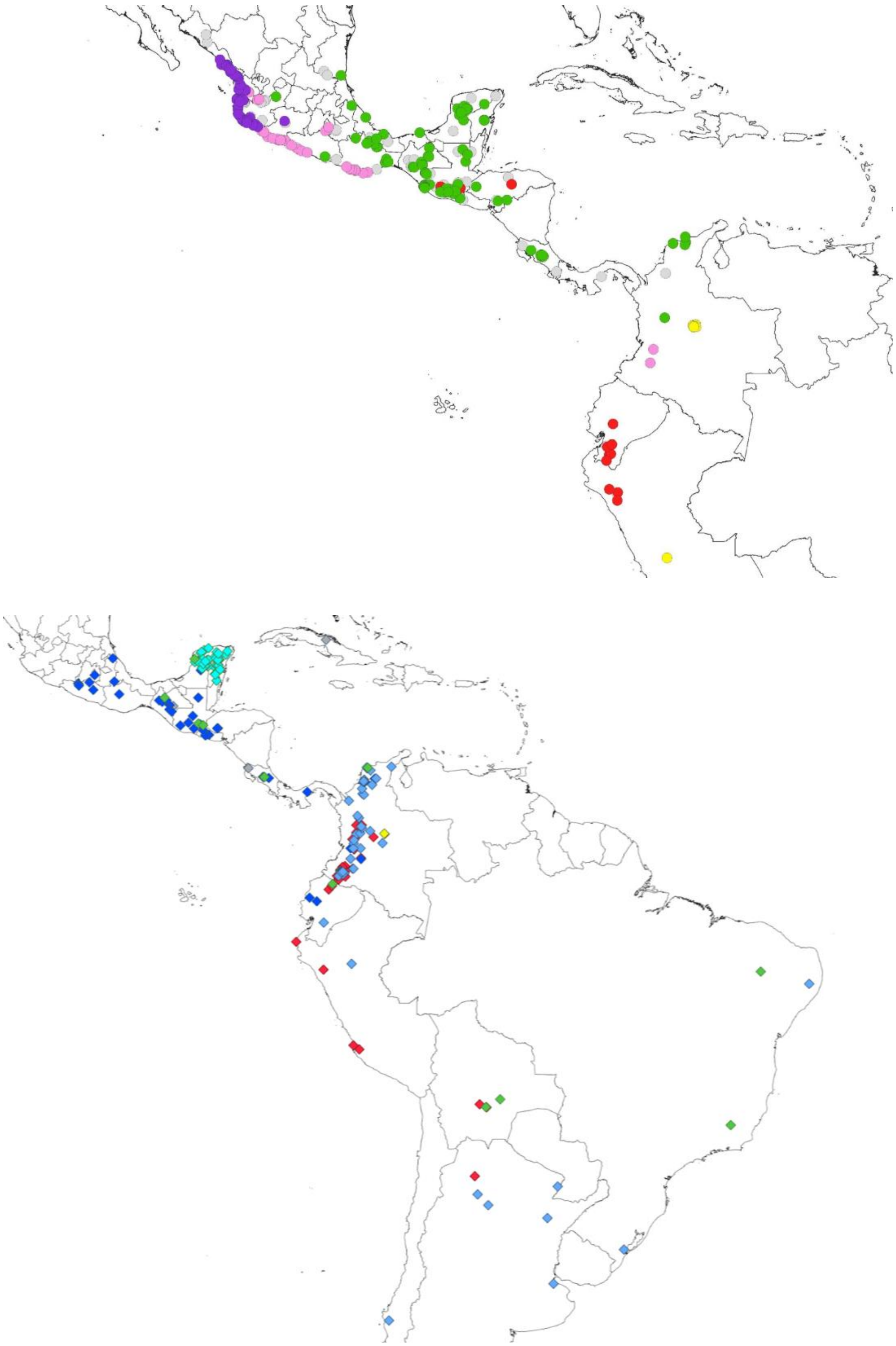

Supplementary Figure 14. Geographic distribution of accessions genotyped in this study, clustered according to the clustering inferred by FineStructure. Wild accessions are shown in the top map and domesticated accessions are shown in the bottom map. Source data are provided as a Source Data file.

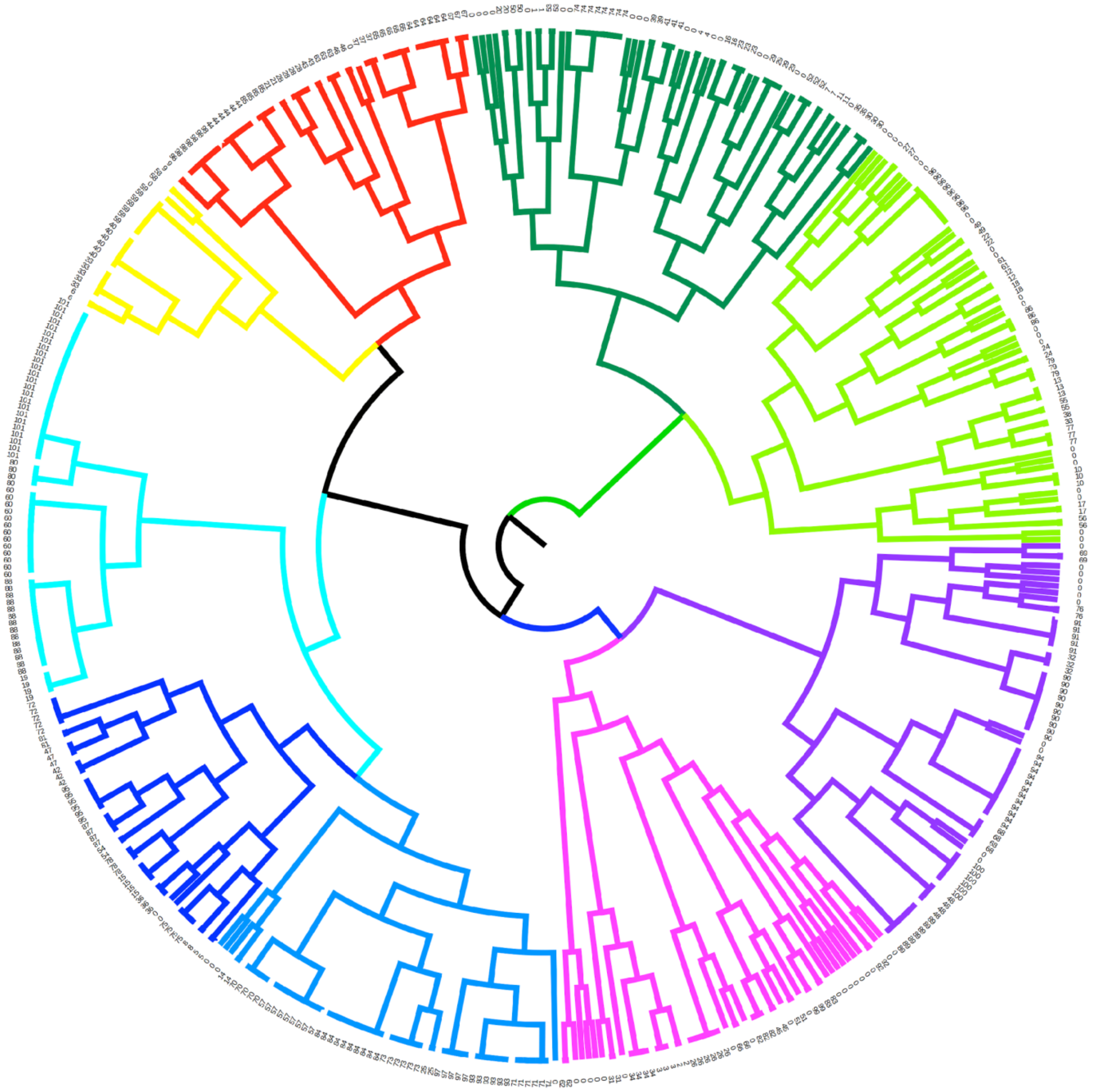

Supplementary Figure 15. Radial clustering of the 482 accessions according to the analysis performed by fineSTRUCTURE. Major gene pools are shown by different colors (purple cluster: wild MI from northern-western Mexico; pink cluster: wild MI from southern-western Mexico; medium blue cluster: domesticated MI from South America; dark blue cluster: domesticated MI from Mexico and Central America (CA); light blue cluster: domesticated MI from Yucatan Peninsula; yellow cluster: AII gene pool; red cluster: AI gene pool; green cluster: MII gene pool from Yucatan, Central America and Colombia; light green cluster: MII gene pool from southern and central Mexico). Population numbers are included for each accession. Source data are provided as a Source Data file.

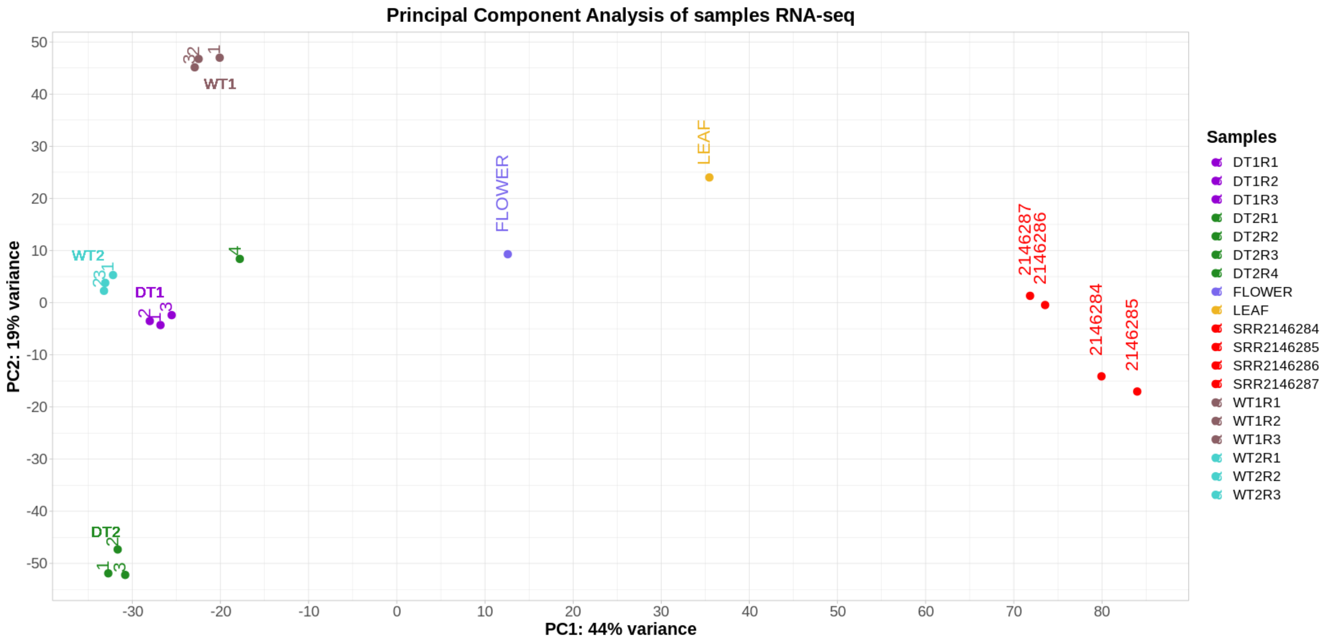

Supplementary Figure 16. Principal component analysis (PCA) of normalized expression values for the complete set of RNA-seq samples used to annotate the reference genome. Based on this analysis, the sample DT2R4 was removed for analysis of differential expression among pod samples between developmental times and between accessions. Source data are provided as a Source Data file.

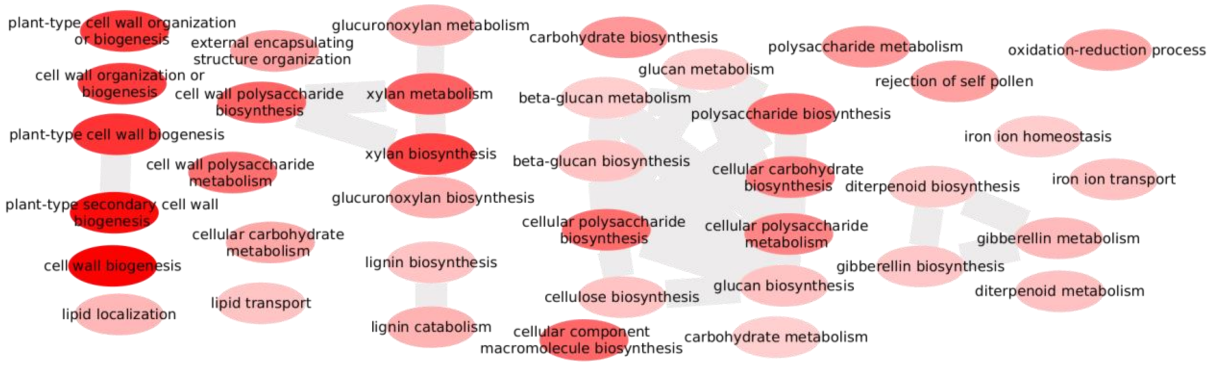

Supplementary Figure 17. Functional enrichment of genes with consistent increased expression between developmental times. Concept map of functional categories enriched in genes with increased expression between the first and the second developmental time for both the wild and the domesticated accessions. Source data are provided as a Source Data file.

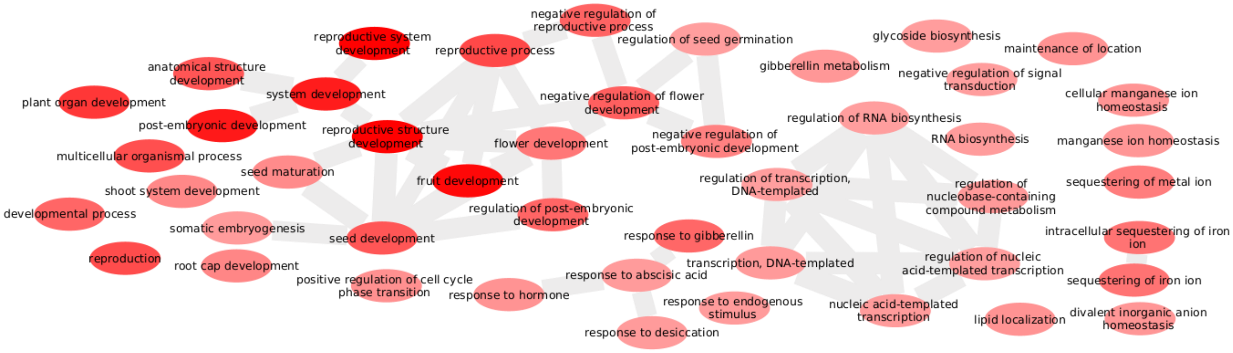

Supplementary Figure 18. Functional enrichment of genes with increased expression in the domesticated accession relative to the wild accession only at the second developmental time. Source data are provided as a Source Data file.

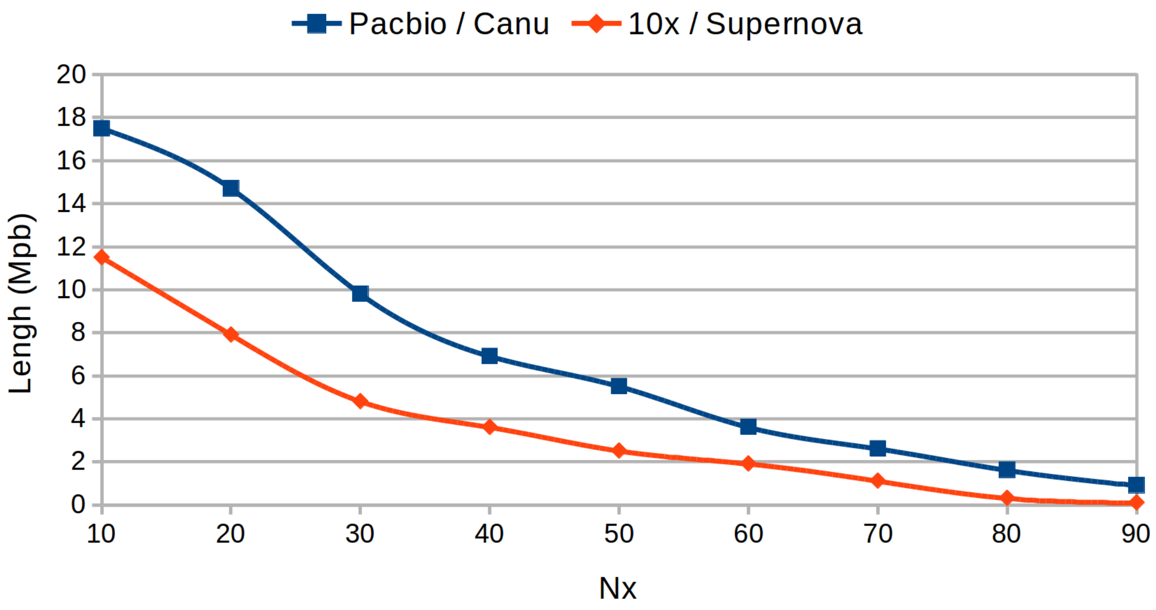

Supplementary Figure 19. Contiguity assessment of genome assemblies. Nx distribution for the assembly obtained from PacBio reads using Canu and the assembly obtained from 10x data using Supernova. Source data are provided as a Source Data file.

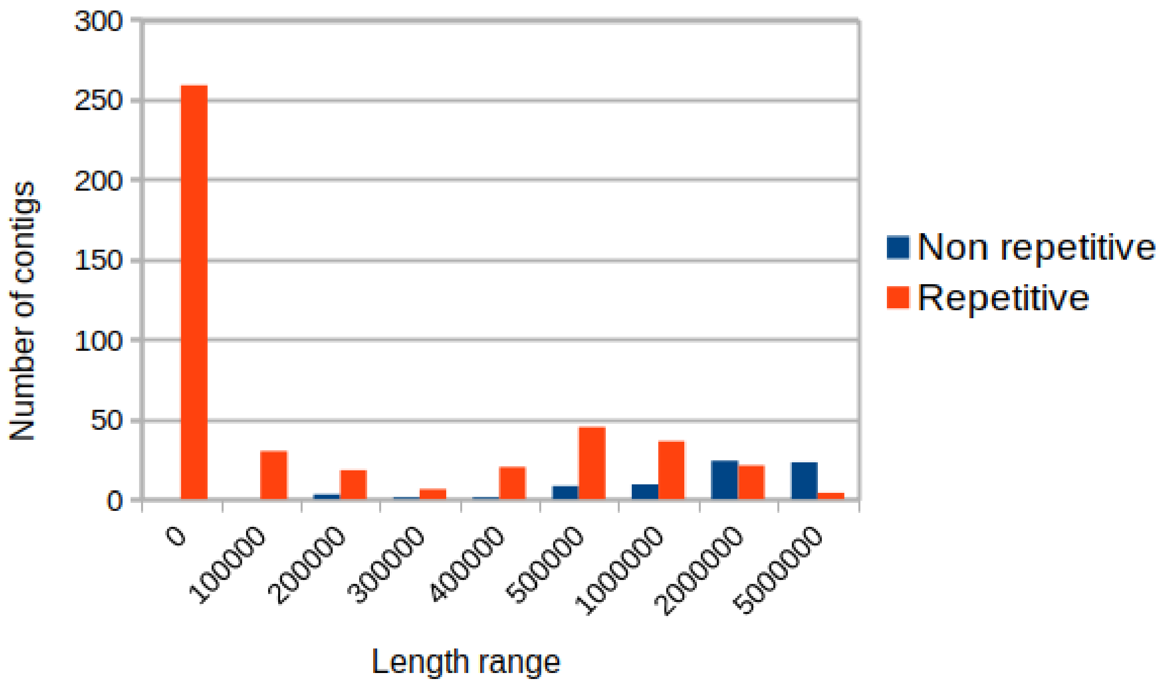

Supplementary Figure 20. Distribution of contig lengths discriminated as repetitive and non repetitive contigs. Source data are provided as a Source Data file.

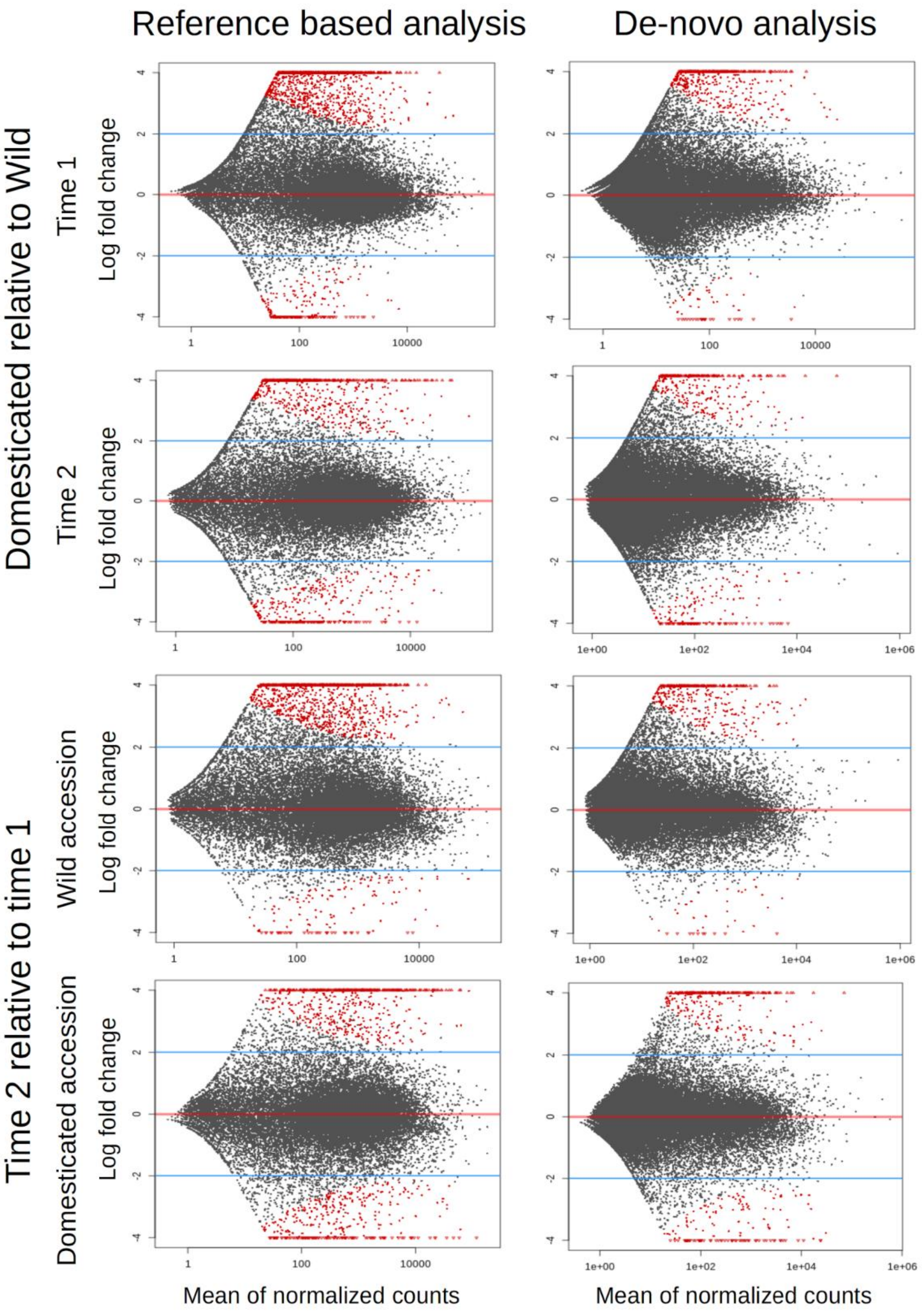

Supplementary Figure 21. Differential expression among RNA-seq samples. Distribution of log fold changes in expression levels inferred from RNA-seq data for the domesticated accession taking the wild accession as a control (two upper panels) and for the developmental stage 2 taking the developmental stage 1 as a control. The red dots indicate significant changes as predicted by DESeq2. Source data are provided as a Source Data file.

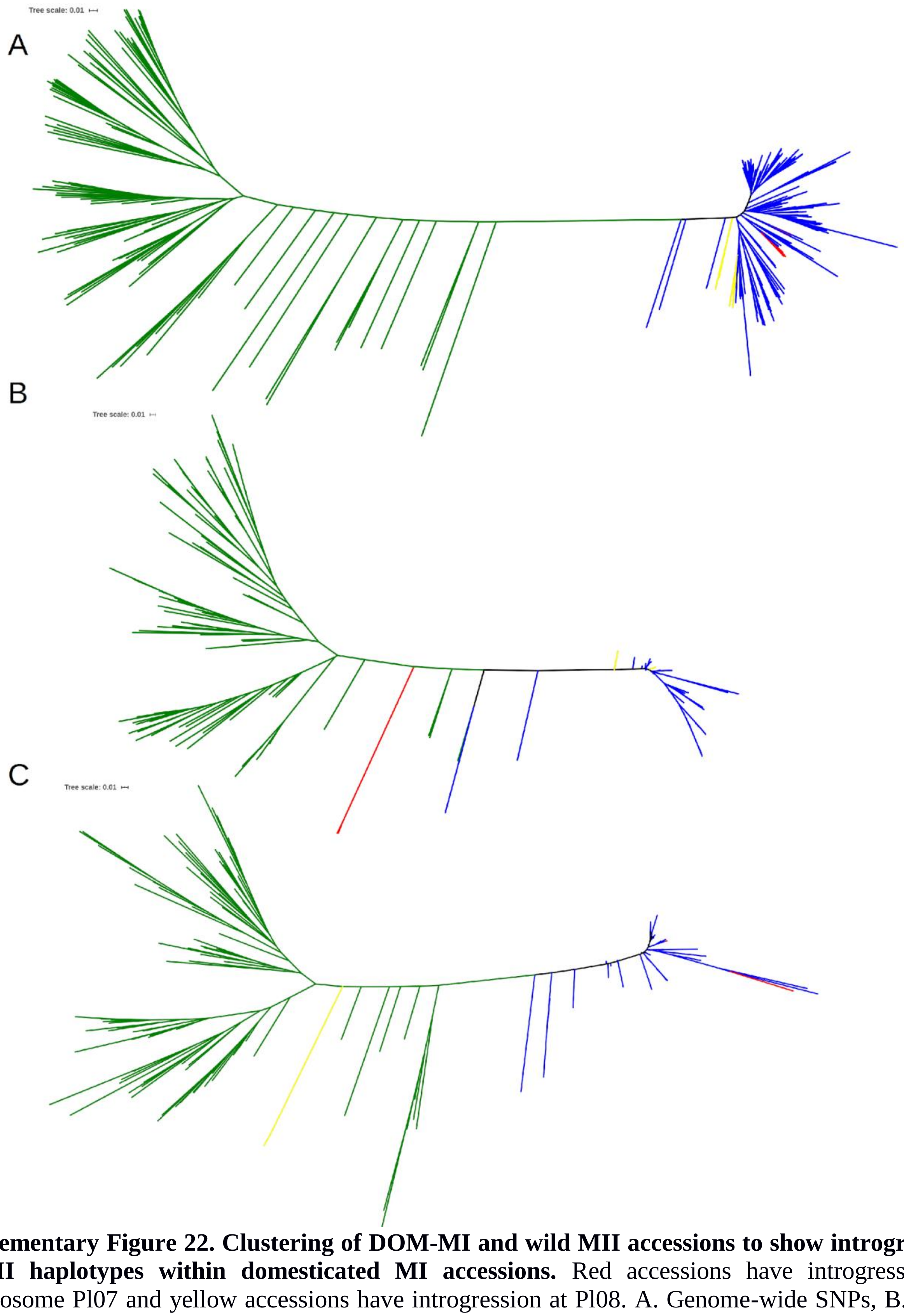

Supplementary Figure 22. Clustering of DOM-MI and wild MII accessions to show introgression of MII haplotypes within domesticated MI accessions. Red accessions have introgression at chromosome Pl07 and yellow accessions have introgression at Pl08. A. Genome-wide SNPs, B. SNPs at chromosome PL07 between 36 and 39 Mbp. C. SNPs at chromosome Pl08 between 4.6 and 7 Mbp. Source data are provided as a Source Data file.
