# Supplementary material for: Comprehensive genomic resources related to domestication and crop improvement traits in Lima bean
Source: Nat Commun. 2021 Jan 29;12:702. doi: 10.1038/s41467-021-20921-1 (PMC7846787; doi:10.1038/s41467-021-20921-1)
Supplement: Supplementary file 3 — Reporting Summary [file 41467_2021_20921_MOESM3_ESM.pdf]

nature research

Corresponding author(s): Maria Isabel Chacón Sanchez
Jorge Duitama

Last updated by author(s): Dec 11, 2020

Reporting Summary

Nature Research wishes to improve the reproducibility of the work that we publish. This form provides structure for consistency and transparency in reporting. For further information on Nature Research policies, see our Editorial Policies and the Editorial Policy Checklist.

Please do not complete any field with "not applicable" or n/a. Refer to the help text for what text to use if an item is not relevant to your study.
For final submission: please carefully check your responses for accuracy; you will not be able to make changes later.

Statistics

For all statistical analyses, confirm that the following items are present in the figure legend, table legend, main text, or Methods section.

n/a	Confirmed	
☐	☒	The exact sample size (*n*) for each experimental group/condition, given as a discrete number and unit of measurement
☐	☒	A statement on whether measurements were taken from distinct samples or whether the same sample was measured repeatedly
☐	☒	The statistical test(s) used AND whether they are one- or two-sided<br>*Only common tests should be described solely by name; describe more complex techniques in the Methods section.*
☒	☐	A description of all covariates tested
☐	☒	A description of any assumptions or corrections, such as tests of normality and adjustment for multiple comparisons
☐	☒	A full description of the statistical parameters including central tendency (e.g. means) or other basic estimates (e.g. regression coefficient) AND variation (e.g. standard deviation) or associated estimates of uncertainty (e.g. confidence intervals)
☐	☒	For null hypothesis testing, the test statistic (e.g. *F*, *t*, *r*) with confidence intervals, effect sizes, degrees of freedom and *P* value noted<br>*Give P values as exact values whenever suitable.*
☐	☒	For Bayesian analysis, information on the choice of priors and Markov chain Monte Carlo settings
☒	☐	For hierarchical and complex designs, identification of the appropriate level for tests and full reporting of outcomes
☒	☐	Estimates of effect sizes (e.g. Cohen's *d*, Pearson's *r*), indicating how they were calculated

Our web collection on statistics for biologists contains articles on many of the points above.

Software and code

Policy information about availability of computer code

Data collection	Conversion of public RNA-seq data to fastq format was performed using the NCBI fastq-dump utility, version 2.9.4
Data analysis	All bioinformatic analyses were performed using the following recognized open source software tools as detailed in the Methods section of the manuscript:<br>fastQC V.0.11.2<br>Trimmomatic v0.36<br>Trinity v.2.4.0<br>BUSCO v2<br>Canu v1.6<br>Bowtie2 v2.3.5<br>Bwa v0.7.17<br>RepeatMasker v4.0.5<br>Maker v2.31.9<br>HISAT2 v2.1.0<br>StringTie v1.3.5<br>DeSeq2 v3.1<br>Salmon v1.2.1<br>Trinotate v3.1.1<br>NCBI Blast v2.10.0<br>HMMer v3.3.1<br>topGO v2.36.0<br>Cytoscape v3.8.1<br>MapGene2Chrom web v2.1

Muscle v3.8.31
MEGA X v10.8.1
iTOL v4.4.2
ASMap v1.0.4
R/qtl v1.44
TASSEL v5
Darwin v6.0.021
Adegenet 2.1.2
Hierfstat v0.5
ANGSD 0.93
STRUCTURE v2.3.4
fineSTRUCTURE v4.1.0

Reference-based analysis of Illumina WGS and GBS data and some of the analysis for comparative and population genomics was performed using our open source software solution NGSEP v3.3.2. Stable releases of NGSEP are available through sourceforge (http://ngsep.sf.net). Live developmet is constantly made available through our github repositories (https://github.com/NGSEP).

For manuscripts utilizing custom algorithms or software that are central to the research but not yet described in published literature, software must be made available to editors and reviewers. We strongly encourage code deposition in a community repository (e.g. GitHub). See the Nature Research guidelines for submitting code & software for further information.

Data

Policy information about availability of data

All manuscripts must include a data availability statement. This statement should provide the following information, where applicable:

- Accession codes, unique identifiers, or web links for publicly available datasets
- A list of figures that have associated raw data
- A description of any restrictions on data availability

Wild and domesticated accessions: accession codes and passport data for the 482 wild and domesticated accessions analyzed in this study are shown in Supplementary File S5 (CIAT's and CICY's accession numbers). Accessions from CIAT can be requested at https://genebank.ciat.cgiar.org. Raw data generated in this study has been deposited in the NCBI Sequence read Archive (SRA) under the Bioproject accession number PRJNA596114 (https://www.ncbi.nlm.nih.gov/bioproject/?term=PRJNA596114). The genome assembled in this study is available at the NCBI assembly database with accession number JAAFYQ000000000 (https://www.ncbi.nlm.nih.gov/assembly/GCA_013389735.1/) The genome assembly and its corresponding annotation are available at Phytozome v13 (https://phytozome-next.jgi.doe.gov/). Other relevant data is available in the supplementary data files. Finally, we reanalyzed RNA-seq data publicly available at NCBI SRA with bioproject accession number PRJNA275266 (https://www.ncbi.nlm.nih.gov/bioproject/?term=PRJNA275266).

Field-specific reporting

Please select the one below that is the best fit for your research. If you are not sure, read the appropriate sections before making your selection.

☒ Life sciences ☐ Behavioural & social sciences ☐ Ecological, evolutionary & environmental sciences

Life sciences study design

All studies must disclose on these points even when the disclosure is negative.

Sample size

Samples for population genetics
A previous study of GBS data on 270 wild and domesticated Lima bean accessions held by the International Center for Tropical Agriculture (CIAT) at Cali, Colombia and the Centro de Investigación Científica (CICY) at Mérida, Yucatán, Mexico (Chacón-Sánchez and Martínez Castillo, 2017) revealed the existence of three gene pools: two Mesoamerican (MI and MII) and one Andean (AI), and interestingly the six wild accessions analyzed from the Andes of central Colombia appeared as a separate group (gene pool AII, although further analyses were needed). This GBS analysis also showed that Mesoamerican landraces clustered within the wild MI gene pool distributed in central-western Mexico and that Andean landraces clustered within the Andean AI gene pool distributed in the Andes of Ecuador-northern Peru. Based on these previous results, we decided to carry out a more comprehensive analysis of the genetic structure of wild and domesticated Lima bean from the Americas and for this purpose we increased the number of accessions to a total of 482 (267 wild and 215 domesticated) with the following criteria. First, we decided to further investigate the genetic structure of wild and domesticated Lima bean from Colombia, a place of interest not only because of the possible existence of a separate wild gene pool in the central Andes of Colombia (gene pool AII) but also because Colombia has been a place of contact of Mesoamerican and Andean landraces since pre-Columbian times. For Colombia, the number of wild and domesticated accessions was increased from 31 (analyzed in 2017) to 97. These accessions included all the wild accessions available at CIAT from gene pool AII (19 in total) and other wild and domesticated accessions from other places in the Andes and the lowlands if northern Colombia. Second, we also decided to further investigate the genetic structure of wild and domesticated Lima bean from Mexico, a country of particular interest for Lima bean evolution because central-western Mexico is the place of origin of the Mesoamerican landraces and also because the Peninsula of Yucatán is currently a place of large diversity of landraces. The sampling from Mexico was increased by means of new field collections made by CICY, especially in states from central-western Mexico, Veracruz and the Peninsula of Yucatán. A total of 104 wild and 33 domesticated accessions were collected. Finally, we included 16 domesticated accessions from the United States because this was a place of early introduction of Lima bean landraces. These landraces were especially selected from the state of Arizona.

Lima bean field collections in Mexico
Wild populations were collected in central-western Mexico, along the Pacific coast from the Chiapas state to Sinaloa, and in the gulf coast in the state of Veracruz. Collection sites were selected to represent the different vegetation types where Lima bean has been reported. Wild

	populations were separated by at least 5 Km from each other. Within each wild population, 20 pods from each of 20 individuals were collected. Seeds were deposited at the germplasm bank at CICY in Mérida, Yucatán, for conservation purposes. Landraces were collected in the states of the Peninsula of Yucatán, Chiapas, Veracruz and Oaxaca, where Lima bean cultivation is currently more common. Landraces were collected from traditional cultivation fields known as milpa and within each cultivation field an effort was made to collect all the different types of landraces present. For the most common landraces, a total of 200-250 seeds were collected per landrace and for the least common landraces, a total of 50-100 seeds were collected per landrace.<br><br>QTL analysis<br>The size of the UC92-UC Haskell (n = 238; P. 36, l. 1049) is above 200 lines, considered to be a minimum standard for QTL analysis. 238 RILs were genotyped, 234 RILs were included for determinacy and seed weight QTL analysis, 127 RILs for days to first flower QTL analysis, and 107 RILs for floral bud cyanogenesis QTL analysis.
Data exclusions	QTL analysis<br>The UC92-UC Haskell populations includes the entire population of viable and fertile F8 lines. Lines that did not have sufficient seed for field planting were excluded in the 2018 field experiment and the days to first flower and cyanogenesis data collection. .<br><br>RNA-seq. One of the replicates of the second developmental time for the domesticated accession was excluded because it did not cluster with the other replicates in the principal components analysis (Supplementry figure 16). PCA is an unsupervised learning procedure used here as a pre-established criterion to evaluate the stability of the replicates . The experiment was repeated to achieve three replicates per condition
Replication	QTL analyses<br>The experiments involving the UC92-UC Haskell populations involved two locations as well as replications within each location. Cyanide measurements for each line of the populations were based on triplicated (biological) samples. For the UC 92 - UC Haskell population, the attempts at two locations and two replications within locations were successful with the exception of the lines without enough seed for field planting, as detailed in the data exclusions section. For the cyanide measurements, the collection of triplicated subsamples were successful across the two locations and replications within locations.<br><br>RNA-seq of pod development<br>For the analysis of differential gene expression related to pod dehiscence, RNA was extracted for the wild and domesticated accession at two pod developmental stages (with three biological replicates each), at the initiation of pod elongation (T1) and before seed filling (T2). Each biological replicate consisted of entire pods harvested from ten different plants.
Randomization	Assignment of lines of the UC92-UC Haskell populations to plots was made on a randomized basis.
Blinding	Lines of the UC92-UC Haskell population are assigned plot numbers (part of the randomization process), which shields the experimenter from the identity of the lines.

Reporting for specific materials, systems and methods

We require information from authors about some types of materials, experimental systems and methods used in many studies. Here, indicate whether each material, system or method listed is relevant to your study. If you are not sure if a list item applies to your research, read the appropriate section before selecting a response.

Materials & experimental systems

n/a	Involved in the study
☒	☐ Antibodies
☒	☐ Eukaryotic cell lines
☒	☐ Palaeontology and archaeology
☒	☐ Animals and other organisms
☒	☐ Human research participants
☒	☐ Clinical data
☒	☐ Dual use research of concern

Methods

n/a	Involved in the study
☒	☐ ChIP-seq
☒	☐ Flow cytometry
☒	☐ MRI-based neuroimaging

This checklist template is licensed under a Creative Commons Attribution 4.0 International License, which permits use, sharing, adaptation, distribution and reproduction in any medium or format, as long as you give appropriate credit to the original author(s) and the source, provide a link to the Creative Commons license, and indicate if changes were made. The images or other third party material in this article are included in the article's Creative Commons license, unless indicated otherwise in a credit line to the material. If material is not included in the article's Creative Commons license and your intended use is not permitted by statutory regulation or exceeds the permitted use, you will need to obtain permission directly from the copyright holder. To view a copy of this license, visit http://creativecommons.org/licenses/by/4.0/
