# Supplementary material for: Comprehensive genomic resources related to domestication and crop improvement traits in Lima bean
Source: Nat Commun. 2021 Jan 29;12:702. doi: 10.1038/s41467-021-20921-1 (PMC7846787; doi:10.1038/s41467-021-20921-1)
Supplement: Supplementary file 4 — Description of Additional Supplementary Files [file 41467_2021_20921_MOESM4_ESM.pdf]

Description of Additional Supplementary Files

Supplementary Data 1. Genomic locations of genes with agronomic annotations inferred by orthology with close species.

Supplementary Data 2. Genes related to resistance to biotic stresses according to orthology with P. vulgaris and functional domains.

Supplementary Data 3. List of accessions genotyped in this study, including latitude, longitude and altitude of the collection site and phenotypic information if available.

Supplementary Data 4. Mapping statistics and number of SNPs genotyped on each sample for diversity analysis.

Supplementary Data 5. Population assignment by STRUCTURE assuming 6 subpopulations and groups assigned by fineSTRUCTURE.

Supplementary Data 6. List of introgressions identified in 52 accessions. The file includes the genomic location, sample id, haplotype similarity score for each major population, the population of origin and the population of the introgressed haplotype.

Supplementary Data 7. List of genes with differential expression values (DEGs) either between developmental stages or between accessions. Functional annotation and expression TPM values are included for each gene.
