# Supplementary material for: Comprehensive genomic resources related to domestication and crop improvement traits in Lima bean
Source: Nat Commun. 2021 Jan 29;12:702. doi: 10.1038/s41467-021-20921-1 (PMC7846787; doi:10.1038/s41467-021-20921-1)
Supplement: Supplementary file 2 — Peer Review File [file 41467_2021_20921_MOESM2_ESM.pdf]

Editorial Note: This manuscript has been previously reviewed at another journal that is not operating a transparent peer review scheme. This document only contains reviewer comments and rebuttal letters for versions considered at *Nature Communications*.

Reviewer #2 (Remarks to the Author):

Overall the authors have dealt with my remaining concerns, only a few minor issues remain.

Perhaps the most important is that the Discussion contains some strong language regarding the diversity conclusions (P 16; It would be nice by the way if the MS had page and line numbers). The conclusions here are much more definite than some of the data in Results seem to indicate would be justified. The use of GBS is somewhat problematic for diversity analysis, as most of the SNPs are usually imputed. I am curious how many measured SNPs in each line were used to base these conclusions, and concerned that the low number of landraces showing introgression signals might just be because of a lack of data, rather than lack of introgression. Nonetheless the GBS data is clearly overall of good quality, and an important point is that this methodology relies completely on the availability of the Lima bean genome, giving an example of the importance of the main result in the manuscript, the genome itself.

Other minor comments:

Abstract: "the present resources" implies the resources are already available, I think the authors mean "the resources presented here".

P2: The P. vulgaris genome alone "produces loss of information". This isn't exactly true and the truth is more important. The use of the P. vulgaris genome prevents Lima researchers from gaining a true picture of genetic diversity, gene expression and causal polymorphism because of reference bias, and also may lead to loss of information when used for analysis in key regions where the two differ.

P3: It is inaccurate to state 10,497 SNP markers were used, as these are not independent "markers". It appears that the number of markers is 522. I am also curious how many of these 522 were observed in each line, versus being missing and imputed. This gives rise to my problems with the introgression statements above.

P4, Fig 1: The legend uses "Phaseolus lunatus" while the text uses "Lima bean". The two should use the same name for the organism.

P4: "SNP density". Note this is the density of SNPs able to be called from GBS, which relies on uniquely mapped sequences; thus it is not a true measure of SNP density or sequence diversity.

Figure 2: Phaseolus lunatus vs Lima bean again. Also legend on parts A and B is hard for me to see the colors representing the samples and impossible if printed out. I would prefer a more saturated, color-blind friendly color scheme, bigger legend and perhaps a different shading on the different samples.

Figure 3: Here, for some reason, we go back to "Lima bean" not "Phaseolus lunatus".

P11: "contained 49 accessions from northern Guatemala, Costa Rica, northern Colombia and few from the Peninsula of Yucatan". It would be good to have a number here rather than "few". It is not clear if the authors mean "few" (in this context, means little or none) or "a few" (a small number).

P14: “Different analysis alternatives” is unclear here and would usually refer to different statistical methods. What I think the authors mean is whether the ratio of gene expression is measured between the stages or accessions?

P15: “Genes with predicted resistance to biotic stresses”. Genes don’t have resistance to biotic stresses, they confer resistance on plants.

P18, Materials and Methods: DNA extraction procedures, and the tissue and stage of plants used for extraction, seem to be missing.

Reviewer #3 (Remarks to the Author):

This revised manuscript is much approved and I appreciate the authors’ efforts on additional data analyses that provide some biological and genetic insights.

Minor:
Last two paragraphs on Page 5: These paragraphs describing QTL mapping should be in a separate section.

Fig. 2c: Please carefully check the inversions shown in this figure, and make sure that the inversions contain no errors introduced such as by insufficient information during the pseudomolecule construction process (e.g., some scaffolds could be non-oriented).

Change “it decays at about 500 kbp” to “it decays to background levels at about 500 kbp” to make it clearer. Also change “basal levels” to “background levels” in the previous sentence.

Comment not directly related to this version of the manuscript:
GWAS (although it’s not related to the current manuscript as the GWAS results have been removed but I think it should be helpful for future related work): I am sorry that the authors misunderstood my comments related to GWAS. I did not mean that GBS data cannot be used for GWAS. Instead, I support using GBS data for GWAS. However, in the previous version of this manuscript, the Manhattan plots showed that only one SNV standing out for most of the association loci (instead of a cluster of SNVs; please see Figs 3-5 of this paper for examples: https://www.nature.com/articles/s41588-018-0266-x). This indicates that the results are suspicious and could be unreliable. This could be due to the low-density of the marker data or inappropriate model used for GWAS. The classical Mixed Linear Model is widely used for GWAS but this does not necessarily mean that the model would fit your data. Trying different models and quality checking such as with Q-Q plots and lambda values are highly recommended.

Reviewer #2 (Remarks to the Author):

Overall the authors have dealt with my remaining concerns, only a few minor issues remain.

Perhaps the most important is that the Discussion contains some strong language regarding the diversity conclusions (P 16; It would be nice by the way if the MS had page and line numbers). The conclusions here are much more definite than some of the data in Results seem to indicate would be justified. The use of GBS is somewhat problematic for diversity analysis, as most of the SNPs are usually imputed. I am curious how many measured SNPs in each line were used to base these conclusions, and concerned that the low number of landraces showing introgression signals might just be because of a lack of data, rather than lack of introgression. Nonetheless the GBS data is clearly overall of good quality, and an important point is that this methodology relies completely on the availability of the Lima bean genome, giving an example of the importance of the main result in the manuscript, the genome itself.

R /
We are glad to know that the answers provided to the comments included in the last review were satisfactory and we thank the reviewer for all his/her suggestions including those present in the current review.

Regarding the potential effect of missing data, we understand that this has been an issue in some manuscripts based on GBS data. However, in our case we performed a conservative filtering to minimize this effect in our downstream analysis. More than 1 million reads were generated for each sample (average 6.8 million) and the high mapping rates (at least 59% and less than 80% for only 20 samples) ensured an adequate number of aligned reads. Although the raw genotype matrix of 116,030 SNPs has about 40% missing data, almost all results related to the diversity analysis were performed on a filtered dataset with 12,398 SNPs, which, among other filters, required at least 450 individuals genotyped for each SNP. The overall missing data rate of this dataset was 5.3%, genotype calls for each sample were present for at least 75% of the SNPs and this percentage is less than 90% for only 6 samples. Moreover, the missing data was not imputed to perform the structure analysis and for general population statistics.

About the low number of landraces showing signals of introgression, we found that about the 10% of the landrace accessions analyzed showed signals of introgression. This is maybe the expected pattern for a predominantly autogamous species as Lima bean. An important aspect to take into account is that in the cultivated field, gene flow from wild populations may be limited through selection that farmers make against hybrids that carry wild traits. Although wild-weedy-crop complexes in Lima bean have not been well-studied, the available data suggest that in some places of the Peninsula of Yucatan gene flow may be asymmetric, predominantly in the direction from crop to wild. Although it is always possible that

a few small introgressions could be identified if the correct genotype calls would somehow be identified to fill the missing data, we do not anticipate a much larger number of introgressions. We improved the methods text (page 25) and added the supplementary data file 4 with the numbers mentioned in this answer.

The only analysis for which imputation was performed was the fineSTRUCTURE analysis because this tool can not handle missing data. Because we had to impute data, we decided to use a less filtered dataset including 28,753 SNPs genotyped in at least 300 accessions. As expected, this dataset has a larger missing data rate of 11%, which was the exact percentage of imputed data. As explained in the results, the results obtained with fineSTRUCTURE were highly consistent with the clustering performed by STRUCTURE and moreover it was consistent at a finer level with the geographical origin of the samples.

Unfortunately, the review is not precise in the exact sentences of the discussion regarding diversity could be more definite than the data included in the results. We performed the following statement by statement analysis:

**Sentence**	**Support with results**
The analysis of this information revealed, at a greater detail, the genetic structure of wild and domesticated Lima beans across their large distribution range in the Americas, (on page 15, first paragraph of the discussion)	Analysis performed by fineSTRUCTURE, compared to Chacon-Sanchez et al 2017.
Genes that confer resistance to biotic stresses on plants show large nucleotide diversity but good correspondence with common bean resistance genes based on orthology predictions and literature. (0n page 15, second paragraph of the discussion)	Diversity analysis of genes related to biotic stresses (Figure 2e)
Accordingly, we found that Andean wild populations are much less diverse than the Mesoamerican ones. In wild common beans, a reduction of diversity was also documented in Andean populations but this time this reduction was attributed to the effect of rare long-distance dispersal events from Mesoamerican populations to the Andes. (on page 16, second paragraph)	Supplementary table 5. Diversity statistics. Average H_E for Wild MI and wild MII is 0.13, whereas for wild AI and wild AII was below 0.05. However, we toned down this statement taking into account that andean populations have a low sample size.

The confirmation of a new wild Lima bean cluster (AII) in the Andes of central Colombia is of great significance because it supports the dispersal of the species from south to north and may represent a new source of alleles not found elsewhere. (on page 16, second paragraph)	STRUCTURE analysis and phaseolin type M8 in supplementary data file 3
With regard to domestication, this study revealed that the domestication bottleneck in Mesoamerica was much more severe than previously thought. (on page 16, second paragraph)	Supplementary table 5. Diversity statistics. Founder effect of 0.55, much larger than that reported in Chacon-Sanchez et al. 2017 (effect of 0.31). However, we toned down this statement using the words “seems to be” instead of “was much”
The genetic diversity that was lost during domestication was apparently hardly re-gained by gene flow from wild to domesticated types, as suggested by the low number of landrace accessions showing signals of introgression. (on page 16, second paragraph)	Results of the introgression analysis (Figure 3d). As detailed below, the 522 loci mentioned in the comment below correspond to the biparental population (not the diversity population) and hence are not related to the analysis of introgressions.
The higher number of samples and genetic markers analyzed in the present study also allowed us to resolve the genetic substructure of the MI gene pool and its relation to geography. Within the MI wild cluster, two subclusters were observed: northern-western Mexico and southern-western Mexico. Also, within the cluster of Mesoamerican MI landraces, three subclusters were detected: Mexico-Central America, South America and the Peninsula of Yucatan. (on page 16, third paragraph)	Results of the fineSTRUCTURE analysis (Figure 3C)
The fact that landraces from Peninsula of Yucatan form a separate subcluster and that their genetic diversity is significantly much lower than other MI landraces, is in agreement with the hypothesis that after its domestication in central-western Mexico, Lima bean was a relatively late adoption of the Mayan communities of the Peninsula of Yucatan. (on page 16, third paragraph)	Supplementary table 5. Diversity statistics. Average H_E for this population was 0.03, the lowest of the entire dataset and lower than MI landraces from other locations (0.07). However, we toned down this statement using the word “relatively” instead of “significantly much”.

Other minor comments:

Abstract: "the present resources" implies the resources are already available, I think the authors mean "the resources presented here".

R/ "the present resources" was changed to "the resources presented here" as suggested by the reviewer. Moreover, the abstract was reduced to 150 words to meet the editorial requirements.

P2: The P. vulgaris genome alone "produces loss of information". This isn't exactly true and the truth is more important. The use of the P. vulgaris genome prevents Lima researchers from gaining a true picture of genetic diversity, gene expression and causal polymorphism because of reference bias, and also may lead to loss of information when used for analysis in key regions where the two differ.

R/ This point was acknowledged in the document by saying that "relying on a *P. vulgaris* genome alone may have consequences for downstream diversity analyses due to reference bias".

P3: It is inaccurate to state 10,497 SNP markers were used, as these are not independent "markers". It appears that the number of markers is 522. I am also curious how many of these 522 were observed in each line, versus being missing and imputed. This gives rise to my problems with the introgression statements above.

R/ We adjusted the text of the result to avoid calling the SNPs "markers". This statement refers to the biparental population (not the diversity population) and hence the 10,497 SNPs identified in this population do not have any relationship with diversity analysis and particularly with the introgression analysis. This population was sequenced at a relatively lower depth and then the overall missing data rate of 21%. These missing data were imputed to build the genetic map and identify QTL.

P4, Fig 1: The legend uses "Phaseolus lunatus" while the text uses "Lima bean". The two should use the same name for the organism.

R/ In the legend of figure 1, "Phaseolus lunatus" was changed to "Lima bean".

P4: "SNP density". Note this is the density of SNPs able to be called from GBS, which relies on uniquely mapped sequences; thus it is not a true measure of SNP density or sequence diversity.

R/ In this part of the text "SNP density" was changed to "observed number of SNPs" for more clarity.

Figure 2: Phaseolus lunatus vs Lima bean again. Also legend on parts A and B is hard for me to see the colors representing the samples and impossible if printed out. I would prefer a more saturated, color-blind friendly color scheme, bigger legend and perhaps a different shading on the different samples.

R/ We followed the suggestions of the reviewer regarding figures 2 a and 2 b. Colors are now more saturated, legends are bigger and numbers are used instead of colors to identify each group. We decided to keep the scientific name *P. lunatus* in this figure because in this figure we show the main results of comparative genomics with close species and then we believe it is appropriate to use scientific names for all compared species in this case.

Figure 3: Here, for some reason, we go back to "Lima bean" not "Phaseolus lunatus".

R/ We chose to keep Lima bean to refer to the species throughout the text (except for figure 2 as explained above). Hence, no changes were made in this figure caption.

P11: "contained 49 accessions from northern Guatemala, Costa Rica, northern Colombia and few from the Peninsula of Yucatan". It would be good to have a number here rather than "few". It is not clear if the authors mean "few" (in this context, means little or none) or "a few" (a small number).

R/ Nine wild accessions from the Peninsula of Yucatan were found in this subcluster, This number was written in the text.

P14: "Different analysis alternatives" is unclear here and would usually refer to different statistical methods. What I think the authors mean is whether the ratio of gene expression is measured between the stages or accessions?

R/ The text was changed accordingly

P15: "Genes with predicted resistance to biotic stresses". Genes don't have resistance to biotic stresses, they confer resistance on plants.

R/ The text was changed accordingly

P18, Materials and Methods: DNA extraction procedures, and the tissue and stage of plants used for extraction, seem to be missing.

R/ We improve the methods text to specify that DNA was extracted from leaf tissue harvested from two-week-old seedlings and in general to provide the details of the DNA extraction protocols used according to each sequencing technology.

Reviewer #3 (Remarks to the Author):

This revised manuscript is much approved and I appreciate the authors' efforts on additional data analyses that provide some biological and genetic insights.

Minor:
Last two paragraphs on Page 5: These paragraphs describing QTL mapping should be in a separate section.

R/ These paragraphs were separated in a new section entitled: "**QTL mapping of agronomic-related traits in Lima bean"**

Fig. 2c: Please carefully check the inversions shown in this figure, and make sure that the inversions contain no errors introduced such as by insufficient information during the pseudomolecule construction process (e.g., some scaffolds could be non-oriented).

R/ We double checked the support information for each of the five structural variation events reported in the analysis. The following table includes the number of contigs (as assembled by Canu using only the PacBio data), the contig lengths, the number of SNPs of the biparental population supporting the orientation and the genetic distance start and end in centimorgans. The contigs supporting the structural variation events were almost perfectly assembled by Canu and the orientation of each case is supported by more than 100 SNPs. The reported length of the event in chromosome Pl03 was updated from 4Mbp to 5Mbp.

**Chr**	**Physical start**	**Physical end**	**Contigs**	**Contig lengths**	**SNPs genetic map**	**Genetic distance start (cM)**	**Genetic distance end (cM)**
Pl10	1	14069759	3	7235537, 6323237, 632489	322	0	26.4
Pl02	1439859	7115077	1	14720824	137	6.6	38.1
Pl03	34231776	39038219	1	6681228	121	73.6	83.8
Pl07	32139752	41554697	1	17147016	308	49.7	60.9
Pl09	2358909	15108160	2	7578912, 6923364	172	30	58.4

Change "it decays at about 500 kbp" to "it decays to background levels at about 500 kbp" to make it clearer. Also change "basal levels" to "background levels" in the previous sentence

R/ The text was changed according to the suggestion.

Comment not directly related to this version of the manuscript: GWAS (although it's not related to the current manuscript as the GWAS results have been removed but I think it should be helpful for future related work): I am sorry that the authors misunderstood my comments related to GWAS. I did not mean that GBS data cannot be used for GWAS. Instead, I support using GBS data for GWAS. However, in the previous version of this manuscript, the Manhattan plots showed that only one SNV standing out for most of the association loci (instead of a cluster of SNVs; please see Figs 3-5 of this paper for examples: https://www.nature.com/articles/s41588-018-0266-x). This indicates that the results are suspicious and could be unreliable. This could be due to the low-density of the marker data or inappropriate model used for GWAS. The classical Mixed Linear Model is widely used for GWAS but this does not necessarily mean that the model would fit your data. Trying different models and quality checking such as with Q-Q plots and lambda values are highly recommended.

R/ We are sorry for this misunderstanding. We will take this suggestion into account for further association analysis, either using the GBS data or future resequencing data.
